# Widespread changes in gene expression accompany body size evolution in nematodes

**DOI:** 10.1093/g3journal/jkae110

**Published:** 2024-05-22

**Authors:** Gavin C Woodruff, John H Willis, Erik Johnson, Patrick C Phillips

**Affiliations:** Institute of Ecology and Evolution, University of Oregon, Eugene, OR 97403, USA; School of Biological Sciences, University of Oklahoma, Norman, OK 73019, USA; Institute of Ecology and Evolution, University of Oregon, Eugene, OR 97403, USA; Institute of Ecology and Evolution, University of Oregon, Eugene, OR 97403, USA; Institute of Ecology and Evolution, University of Oregon, Eugene, OR 97403, USA

**Keywords:** *Caenorhabditis*, *C. elegans*, transcriptomics, body size, evo-devo

## Abstract

Body size is a fundamental trait that drives multiple evolutionary and ecological patterns. *Caenorhabditis inopinata* is a fig-associated nematode that is exceptionally large relative to other members of the genus, including *Caenorhabditis elegans*. We previously showed that *C. inopinata* is large primarily due to postembryonic cell size expansion that occurs during the larval-to-adult transition. Here, we describe gene expression patterns in *C. elegans* and *C. inopinata* throughout this developmental period to understand the transcriptional basis of body size change. We performed RNA-seq in both species across the L3, L4, and adult stages. Most genes are differentially expressed across all developmental stages, consistent with *C. inopinata*'s divergent ecology and morphology. We also used a model comparison approach to identify orthologues with divergent dynamics across this developmental period between the 2 species. This included genes connected to neurons, behavior, stress response, developmental timing, and small RNA/chromatin regulation. Multiple hypodermal collagens were also observed to harbor divergent developmental dynamics across this period, and genes important for molting and body morphology were also detected. Genes associated with transforming growth factor β signaling revealed idiosyncratic and unexpected transcriptional patterns given their role in body size regulation in *C. elegans*. This widespread transcriptional divergence between these species is unexpected and maybe a signature of the ecological and morphological divergence of *C. inopinata*. Alternatively, transcriptional turnover may be the rule in the *Caenorhabditis* genus, indicative of widespread developmental system drift among species. This work lays the foundation for future functional genetic studies interrogating the bases of body size evolution in this group.

## Introduction

The size of an organism is both conspicuous and central to its way of life. Life-history strategies are intimately tied to body size; for instance, as larger organisms tend to develop more slowly (McMahon and Bonner 1983; Calder 1984), body size underlies trade-offs between maturation time and larval survival (Stearns 1992). Body size dictates the kinds of organisms one directly interacts with as well as the nature of those interactions (Peters 1983; Calder 1984). Moreover, the physical space an organism occupies dictates the scale of its influence on the environment (i.e. body size correlates with home range: A single bacterial cell's spatial sphere of influence is vastly different from that of a single blue whale) (Peters 1983; Calder 1984). As a consequence, the diversity of body sizes in the natural world is immense [21 orders of magnitude (McMahon and Bonner 1983)] and obvious. A satisfying explanation of diversity will then require an account of the causes (both proximate and ultimate) of body size diversity.

**Fig. 1.**
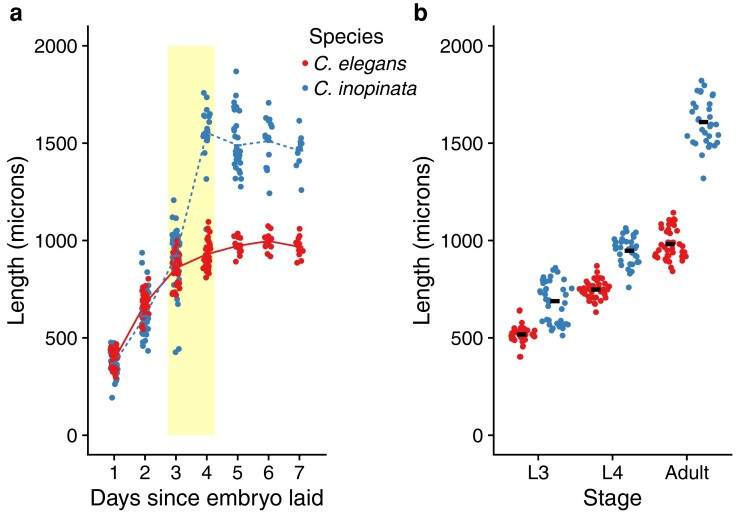
Motivation: *C. inopinata* reveals an increased growth rate during the larval-to-adult transition. a) Size over time. The yellow box covers the L4-adult transition in *C. inopinata*, where the growth rate in *C. inopinata* rapidly increases. Lines connect means. b) Size at the L3, L4, and adult stages. Sina plots are strip charts with points taking the contours of a violin plot. Horizontal bars represent means. Data from Woodruff *et al.* (2018).

One approach toward understanding how body sizes change is to study closely related organisms with divergent body sizes where the genetic traces of the bases of evolutionary change are still detectable. The nematode genus *Caenorhabditis* is well-positioned to address this problem—*Caenorhabditis elegans* is a model system with sophisticated genetic tools (Corsi *et al.* 2015), and its sister species, *Caenorhabditis inopinata*, has rapidly evolved a much larger body size [being 64–200% longer in body length (Kanzaki *et al.* 2018; Woodruff *et al.* 2018)]. In a previous study, we showed that this body size difference largely occurs due to postembryonic events during the larval-to-adult transition (Woodruff *et al.* 2018) (Fig. 1). Additionally, we showed that this difference was not due to changes in cell number nor epidermal ploidy (Woodruff *et al.* 2018). We then concluded that changes in cell size upon maturation were the major driver of body size divergence in this system (Woodruff *et al.* 2018).

Another advantage of the *Caenorhabditis* system is the vast body of background knowledge associated with *C. elegans* (Corsi *et al.* 2015). A number of body size mutants have been isolated in this system, providing a wealth of genetic fodder for evolutionary hypotheses regarding the bases of body size divergence in *C. inopinata*. For instance, multiple genes in the transforming growth factor β (TGF-β) pathway reveal body size phenotypes when perturbed with mutation or RNAi (Savage-Dunn and Padgett 2017). Moreover, there are dose-dependent responses of TGF-β signaling factors on body size in *C. elegans* (Morita *et al.* 1999; Suzuki *et al.* 1999). Thus, one hypothesis for the evolution of body size in *C. inopinata* would be the modulation of TGF-β pathway activity via changes in gene expression. Likewise, multiple cuticle collagens also regulate body size in *C. elegans* (Madaan *et al.* 2018; Johnstone 2000); changes in their copy number or expression could also underlie body size evolution in *C. inopinata*. Indeed, hundreds of genes in *C. elegans* have been shown to influence body size (Schindelman *et al.* 2011), and all of them represent hypothetical drivers of body size divergence in *C. inopinata*. To test these hypotheses, we performed RNA-seq in *C. elegans* and *C. inopinata* across the L3-Adult transition to find genes with divergent developmental dynamics that are potentially connected to the evolution of body size.

## Materials and methods

### Strains, culture conditions, and developmental staging

*C. inopinata* NKZ35 (Kanzaki *et al.* 2018) and *C. elegans fog-2(q71)*JK574 (Schedl and Kimble 1988) were used for this study. Animals were maintained on Nematode Growth Media (with 3.2% agar) seeded with the food source *Escherichia coli*OP50-1 at 25°C. Animals were synchronized by allowing 15 adult *C. elegans* (or 70 adult *C. inopinata*) gravid females to lay embryos for 3 h. Fifteen such synchronization plates were established for each species. After egg-laying, plates were monitored to ensure 100–200 embryos per plate were laid. *C. inopinata* mixed-sex populations were washed off of plates in M9 buffer at 48 (L3), 66 (L4), and 83 (adult) hours after embryos were laid. *C. elegans* mixed-sex populations were washed off of plates in M9 buffer at 23 (L3), 30 (L4), and 40 (adult) hours after embryos were laid. Before isolating populations, plates were examined to ensure nematodes exhibited morphology consistent with their presumptive developmental stage. After washing, worm concentrations were measured to ensure each tube contained 100 nematodes. Five samples were isolated per species per developmental stage. Nematodes were then resuspended in 250 μl of TRIzol, flash-frozen in liquid nitrogen, and stored at −80°C.

### RNA isolation, library preparation, and sequencing

Nematodes in TRIzol were subjected to 10 freeze–thaw cycles in liquid nitrogen for tissue disruption. RNA was then isolated with the Qiagen RNeasy kit and resuspended in 15 μl of RNAse-free water. We used 100 ng of total RNA for mRNA extraction and Illumina library preparation using the KAPA mRNA Hyper prep kit (KK8580). Samples were sequenced on the Illumina HiSeq 4000 platform at the University of Oregon (https://gc3f.uoregon.edu/).

### Read processing, transcript abundance, orthology, and domain inference

Read quality was evaluated with FastQC (with default options) (Andrews 2010). Reads were then demultiplexed with Stacks process_shortreads (with options “-q -c -r –index_null”) (Rochette *et al.* 2019). The barcode for one sample (a *C. inopinata* L4 sample) was not recovered, and this sample could not be included in the analysis. *C. inopinata* (Kanzaki *et al.* 2018) *C. elegans* (Yoshimura *et al.* 2019), *C. briggsae* (Stein *et al.* 2003), *C. nigoni* (Yin *et al.* 2018), and *C. remanei* (Teterina *et al.* 2023) genome assemblies, annotations, mRNA FASTA files, and protein FASTA files were retrieved from WormBase ParaSite (Howe *et al.* 2017). *C. elegans* and *C. inopinata* mRNA FASTA files then were filtered to remove all alternative splice variants except the largest isoform of each gene. These mRNA files were used to generate indices with salmon index (with default options) (Patro *et al.* 2017). *C. inopinata* and *C. elegans* RNA-seq reads were mapped to their respective reference transcriptomes and transcript abundances inferred with salmon quant (with options “-l A -*p* 8 –validateMappings –gcBias”) (Patro *et al.* 2017). One *C. elegans* L3 sample revealed a low number of reads that mapped to the reference (Supplementary Table 1). This sample was then excluded from downstream analyses.

All *Caenorhabditis* protein files were filtered to remove alternative splice isoforms (while retaining the largest isoform per gene), and these files were prepared for the OrthoFinder software (with the command “orthofinder -op -S blast −f”) (Emms and Kelly 2019). All pairwise query-database whole-protein searches were performed with blastp (with options “-outfmt 6 -evalue 0.001”) (Camacho *et al.* 2009). Orthologues were identified with OrthoFinder (with options “-S blast -M msa -a 10”) (Emms and Kelly 2019). One-to-one orthologues among *C. elegans* and *C. inopinata* were extracted from the output file “Orthogroups.GeneCount.tsv,” and these defined the 10,718 genes used for downstream analyses of RNA-seq data. This file was also used to identify: *C. inopinata*-specific genes not placed into an orthogroup (“*C. inopinata* orphan”); genes found only in *C. inopinata* that clustered into orthogroups containing only *C. inopinata* genes (“*C. inopinata*-specific multi-copy”); genes present in multiple *Caenorhabditis* species aside from *C. elegans* (“Multi-*Caenorhabditis C. elegans*-absent”); and genes present in both *C. elegans* and *C. inopinata* while part of orthogroups with more than one gene copy in some species (“Multi-copy orthogroup present in both species”). InterProScan (version 5.65–97.0; with default options) (Jones *et al.* 2014) was used to find domains in the protein sequences used in this work. Additionally, a separate analysis using all 21,472 *C. inopinata* protein-coding genes was performed to include genes that are not one-to-one *C. inopinata*–*C. elegans* orthologues. For this particular analysis, *C. elegans* samples were not included, and only *C. inopinata* samples and genes were considered.

### Differential gene expression analyses

Differential gene expression analyses and modeling were performed with DeSeq2 (Love *et al.* 2014), implemented in R (R Core Team 2024). For the single-copy orthologue analysis, only one gene had a count less than one across all samples and was excluded from downstream analyses (leaving 10,717 single-copy orthologues). For the data set including all *C. inopinata* genes, 1,803 genes had a count less than one across the *C. inopinata* samples (leaving 19,669 genes for this analysis). DeSeq2 fits a generalized linear model of raw gene counts following a negative binomial distribution with a given mean and dispersion for each gene (Love *et al.* 2014); log_2_ fold change coefficients are estimated for each sample type (the DeSeq2 function was called with default arguments). For single-copy orthologues, principal component analysis performed on regularized log-transformed counts (with the *prcomp()* function in R with default options) revealed clustering among groups (Supplementary Figs. 1 and 2). For single-copy orthologues, DeSeq2 was also used to perform Wald tests for each gene among *C. elegans* and *C. inopinata* at each of the 3 developmental stages (with the function “results”) (Love *et al.* 2014). Additionally, models including a species–stage interaction term (“∼ Species + Stage + Species:Stage”) were fitted and likelihood ratio tests performed [with the reduced model (“∼ Species + Stage“)] in DeSeq2; in this case, the interaction term gives the estimated difference between the stage effect (across the L4-adult stages) for *C. inopinata* and the stage effect for *C. elegans* (Love *et al.* 2014). For the analysis with all *C. inopinata* genes, models including a stage term “∼ Stage + Date RNA prepared” were fitted and likelihood ratio tests performed (with the reduced model “∼ Date RNA prepared”) in DeSeq2. Here, model coefficients for each gene were extracted to detect genes with positive and negative transcriptional trajectories across development.

Weighted gene co-expression network analyses were performed with the normalized gene counts generated above through the Weighted Gene Correlation Network Analysis (WGCNA) package in R (Langfelder and Horvath 2008). Soft-thresholding power values were selected with the *pickSoftThreshold()* function (options corFnc = cor; networkType = “signed”). Model coefficients were plotted with soft power thresholds to select such values for WGCNA. Signed co-expression networks were inferred with the *blockwiseModules()* function (options maxBlockSize = 5,000; TOMType = “signed”; power = 12; randomSeed = 1234). Linear models were fit on each module to find modules characterized by genes with significant transcriptional change over development (using the *limma* package *lmFit()* function with default parameters; the model formula “∼ Stage” was used). Additionally, clusters were also inferred with hierarchical clustering for the dataset including all *C. inopinata* genes. This was performed with the *hclust()* function in R (options method=”complete”) (Murtagh and Contreras 2012). The resultant tree was split into 20 clusters with the *cutree()* function (options *k* = 20). Linear models were fit on each cluster to find those characterized by genes with significant transcriptional change over development. As most clusters revealed significant developmental dynamics (Supplementary Fig. 6), six clusters with the most striking dynamics were chosen for visualization (Supplementary Fig. 7). Orthologue type counts and domain counts across modules, clusters, or developmentally dynamic genes were compared with whole-genome counts with χ^2^ tests (*chi.sq()* function in R with default options).

*P*-values were corrected for multiple tests with the Holm method (Holm 1979) or Benjamini and Hochberg method (Benjamini and Hochberg 1995). Such correction was implemented in all cases where multiple hypothesis tests were performed. For principal component analysis (PCA) and data visualization, counts were regularized log_2_ transformed (function “rlog” with option “blind = FALSE”) (Love *et al.* 2014). Computational workflows, statistical analyses, and data have been deposited in GitHub (https://github.com/gcwoodruff/inopinata_developmental_transcriptomics_2023/).

### Gene set enrichment analyses

For the single-copy orthologue analysis, genes with significant species–stage interactions (Supplementary Table 2) were rank-ordered by the interaction term and the top 10% (“positive interactions”; Supplementary Table 3) and bottom 10% (“negative interactions”; Supplementary Table 4) of these genes were extracted for ontology analyses. These lists were used as the input for the WormBase “Gene Set Enrichment Analysis” tool (Angeles-Albores *et al.* 2016, 2018) (Fig. 4). This reveals enrichment of WormBase Tissue (Lee and Sternberg 2003), WormBase Phenotype (Schindelman *et al.* 2011), and Gene Ontology (Ashburner *et al.* 2000) terms in the input gene list compared to all *C. elegans* genes. Similar gene set enrichment analyses were performed with *C. inopinata* orthologues harboring high numbers of species-specific amino acid replacements (see below).The same significant species–stage interaction lists were used as input for the WormExp tool (Yang *et al.* 2016) (Fig. 5), which compares the input list with 2,953 gene lists from previous *C. elegans* -omics experiments. Kyoto Encyclopedia of Genes and Genomes (KEGG) pathway over-representation analyses were performed with the WebGestalt tools (Wang *et al.* 2017).

### Species-specific amino acid replacements

Before performing RNA-seq studies, species-specific amino acid replacements across the *Caenorhabditis* genus were considered. Here, protein sets from 14 species of the Elegans group of *Caenorhabditis* were retrieved. These species included: *C. kamaaina*, *C. inopinata*, *C. elegans*, *C. brenneri*, *C. doughertyi*, *C. tropicalis*, *C. wallacei*, *C. latens*, *C. remanei*, *C. briggsae*, *C. nigoni*, *C. sinica*, *C. zanzibari*, and *C. tribulationis* (THE C. ELEGANS SEQUENCING CONSORTIUM 1998; Stein *et al.* 2003; Fierst *et al.* 2015; Kanzaki *et al.* 2018; Yin *et al.* 2018; Stevens *et al.* 2019; Teterina *et al.* 2020). OrthoFinder (version 1) (Emms and Kelly 2019) was used to prep protein sets for all-by-all blastp (options -f -ob). Blastp (version 2.2.30; options -outfmt 6 -evalue 0.001 -num_threads 8) (Camacho *et al.* 2009) then was used to identify similar sequences among protein sets. OrthoFinder was then used to find orthologous groups among the 14 species (option -b); 2,793 single-copy orthologues were identified. Single-copy orthologues were extracted and aligned with MAFFT (options –auto) (Katoh *et al.* 2002). Alignments were trimmed with trimal (options -gt 1) (Capella-Gutiérrez *et al.* 2009). Custom bash and python scripts were then used to identify and count species-specific amino acid replacements (scripts can be found at https://github.com/gcwoodruff/inopinata_developmental_transcriptomics_2023/tree/main/G3_revisions_1/species-specific_amino_acid_replacements). For the species-specific amino acid replacement results reported here, only alignments >19 amino acids in length were considered (leaving 2,767 alignments).

### Expression of transposon-aligning genes

The TransposonPSI database [(Langfelder and Horvath 2008); file “transposon_db.pep”] was used to generate a BLAST database to which the *C. elegans* and *C. inopinata* protein sets were queried with blastp (options “-outfmt 6 -evalue 0.005”) (Camacho *et al.* 2009). *C. inopinata* proteins that aligned to this database (that also did *not* align with any *C. elegans* proteins) were classified as “transposon-aligning” proteins. These were used to compare the 2 types of genes (those that do and do not align transposons) considering the whole *C. inopinata* gene set in Fig. 7 (irrespective of homology with *C. elegans* genes).

The R packages “airway” (Himes *et al.* 2014), “tximport” (Soneson *et al.* 2016), “DESeq2” (Love *et al.* 2014), “PoiClaClu” (Witten 2019), “ggplot2” (Wickham 2016), “ggforce” (Pedersen 2024a), “cowplot” (Wilke 2024), “patchwork” (Pedersen 2024b), “reshape2” (Wickham 2007), “lemon” (Edwards 2024), “GGally” (Schloerke et al. 2024), and “tidyr” (Wickham et al. 2024) were used for this study. Details of computational workflows have been deposited in GitHub (https://github.com/gcwoodruff/inopinata_developmental_transcriptomics_2023/).

## Results

### Most genes are differentially expressed and exhibit divergent dynamics among species

The length difference between *C. elegans* and *C. inopinata* increases dramatically during the L4-adult transition (Woodruff *et al.* 2018) (Fig. 1). To understand the transcriptional basis of body length divergence, we performed RNA-seq on populations of both species at the L3, L4, and adult stages. Differences in reproductive mode among species were accounted for by using *C. elegans fog-2(q71)* animals. This is a *C. elegans*-specific gene encoding an F-box protein implicated in germ-line sex determination (Nayak *et al.* 2004). Hermaphrodites homozygous for the *fog-2(q71)* genotype are unable to produce sperm, and this mutation effectively causes *C. elegans* to behave as a female/male species (with obligate outcrossing and a 50:50 sex ratio) (Schedl and Kimble 1988). This allowed both species to harbor mixed-sex populations and facilitated transcriptomic comparisons.

**Fig. 2.**
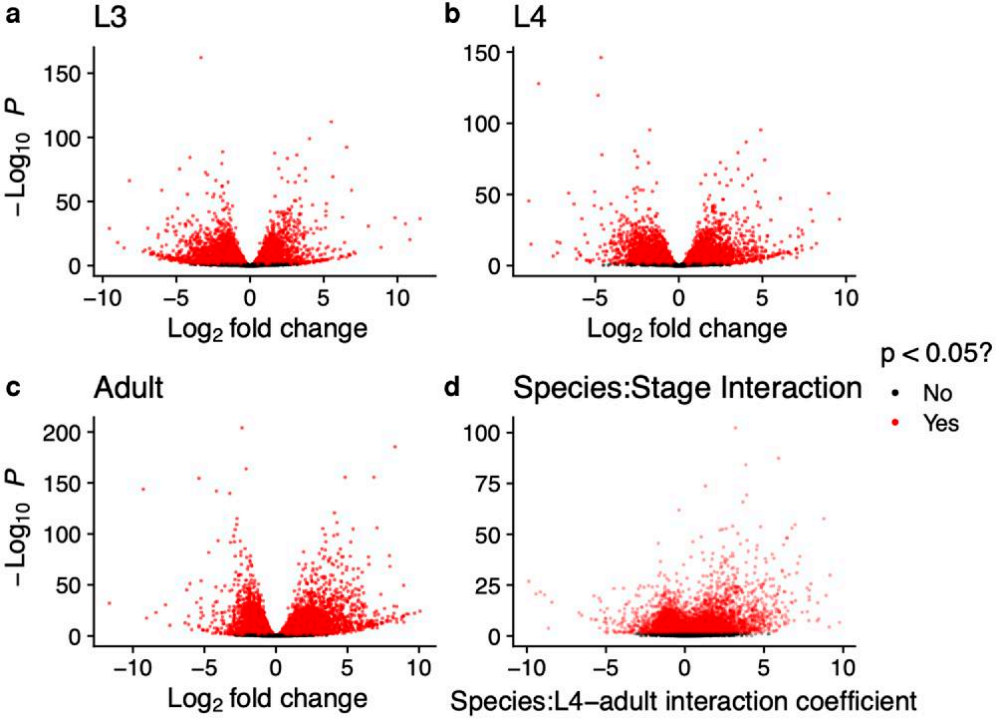
Conventional (a–c) and interaction (d) volcano plots. Differential gene expression between *C. elegans* and *C. inopinata* at the L3 (a), L4 (b), and Adult (c) stages. Also plotted are species:stage interaction coefficients by –log_10_(*P*) (d). All *P*-values were corrected for multiple testing (Holm method; see Materials and methods).

Most genes were differentially expressed at all developmental stages among 10,817 single-copy orthologues. 57, 55, and 66% of these genes were differentially expressed between *C. elegans* and *C. inopinata* at the L3, L4, and adult stages, respectively (Wald Test Holm-adjusted *P* < 0.05; Fig. 2a–c; Supplementary Tables 5–7). To identify genes with divergent dynamics across the key developmental window of interest (the L4-adult transition; Fig. 1), we used a model comparison approach to identify genes with significant species–stage interactions with respect to this developmental window (see Materials and methods). This likewise revealed about two-thirds of the single-copy orthologues (67%; 7,204/10,817) exhibit divergent dynamics across this period (Likelihood Ratio Test Holm-adjusted *P* < 0.05; Fig. 2d; Supplementary Table 2). Thus, not only are most genes differentially expressed at any given developmental stage, most genes reveal differing dynamics across developmental stages.

### Genes with highly divergent dynamics tend to have behavioral, cuticular, germline, and stress–response functions

**Fig. 3.**
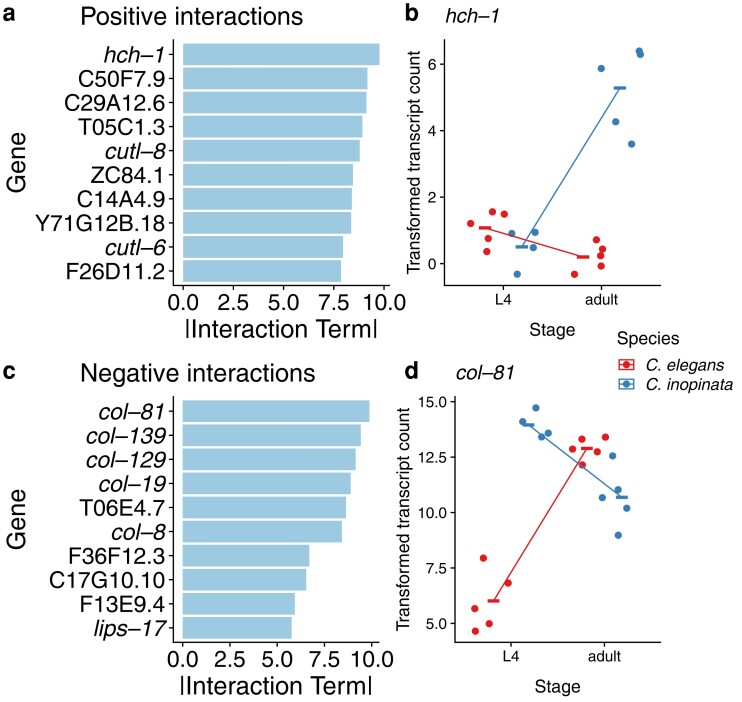
Top genes with divergent dynamics. Genes with the top positive (a, b) and negative (c, d) species–stage interaction terms are plotted. The transcriptional counts (regularized log-transformed) of *hch-1* (b) and *col-81* (c) across the L4-Adult transition are also plotted.

To understand the kind of genes exhibiting divergent developmental dynamics among species, ontology enrichment analyses were performed. However, as most genes revealed significant species–stage interactions (Fig. 2d; Supplementary Table 2), the top 10% (Supplementary Table 3) and the bottom 10% (Supplementary Table 4) of these genes as ranked by species–stage interaction coefficient were used for enrichment analyses (Fig. 3). These defined the “Positive Interactions” list (720 genes; Supplementary Table 2) and the “Negative Interactions” list (720 genes; Supplementary Table 3). As expected, genes with high species–stage interaction coefficients reveal genes whose expression increases across the L4-adult developmental window in *C. inopinata* but decrease in *C. elegans* (Fig. 3a). Genes with low species–stage interaction coefficients reveal the opposite pattern—such genes decrease in expression across this window in *C. inopinata* while increasing in *C. elegans* (Fig. 3b). Notably, among the genes with the 10 lowest species–stage interaction coefficients, 5 encode cuticle collagens (Fig. 3b).

**Fig. 4.**
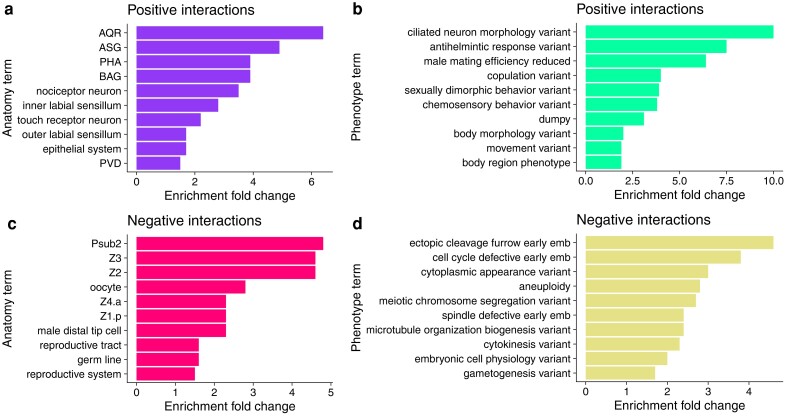
WormBase ontology enrichment among genes with divergent developmental dynamics. Significantly enriched WormBase anatomy (a, c) and WormBase phenotype (b, d) terms are plotted. Genes with positive (a–b; Supplementary Table 1 Sheet 3) and negative (c–d; Supplementary Table 1 Sheet 4) species–stage interactions across the L4-Adult transition were used for these analyses.

**Fig. 5.**
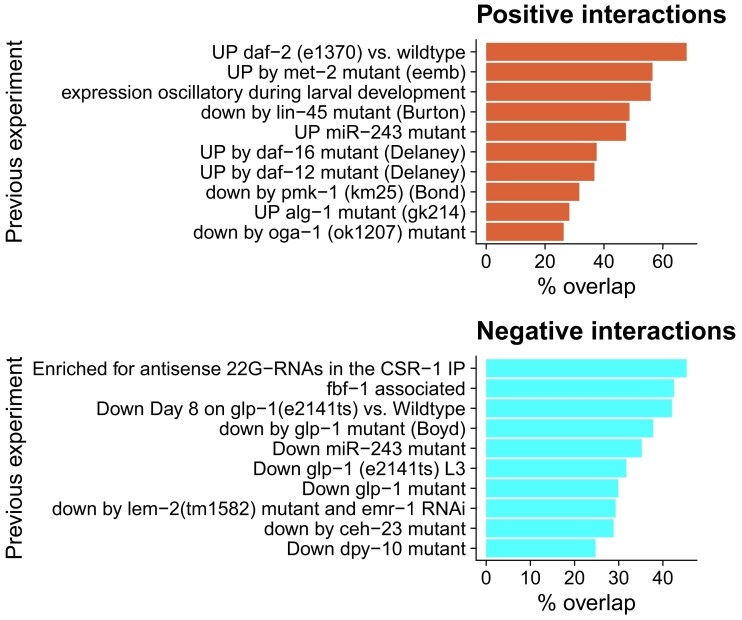
WormExp enrichment among genes with divergent developmental dynamics. The top 10 previous experiments with significantly overlapping gene lists are plotted. Genes with positive (top panel; Supplementary Table 1 Sheet 3) and negative (bottom panel; Supplementary Table 1 Sheet 4) species–stage interactions across the L4-Adult transition were used for these analyses.

Both lists were analyzed with the WormBase Gene Enrichment tool (Angeles-Albores *et al.* 2016, 2018), which compares the frequencies of *C. elegans*-specific tissue and phenotype ontology terms associated with an observed gene list with those expected in the entire *C. elegans* gene set. The genes with positive interactions were enriched for genes expressed in neurons (Fig. 4a), and genes with neuronal and behavioral phenotypes upon perturbation (Fig. 4b). Additionally, such genes were also enriched for morphological phenotypes such as “dumpy” and “body morphology variant” (Fig. 4b). Genes with negative interactions were enriched for germline, somatic gonad, and early embryonic cell expression (Fig. 4c) as well as early-embryo and germline phenotypes (Fig. 4d). In addition to species-specific tissue and phenotype ontologies, the 2 lists were also analyzed with the WormExp tool (Yang *et al.* 2016) (Fig. 5). This tool compares a gene list from a *C. elegans* genomics study and compares it with a curated collection of such gene lists from previous *C. elegans* experiments; this tool identifies lists with an unexpected degree of overlap. Genes with positive species–stage interactions revealed high overlap with previous *C. elegans* experiments examining stress–response (Zarse *et al.* 2012; Bond *et al.* 2014; Burton *et al.* 2017; Delaney *et al.* 2017), small RNAs (Corrêa *et al.* 2010; Zisoulis *et al.* 2010; Padeken *et al.* 2019), and the molting cycle (Hendriks *et al.* 2014) (Fig. 5). Conversely, genes with negative species–stage interactions likewise revealed overlap with studies regarding stress-response (Rohlfing *et al.* 2010; Chang *et al.* 2017) and small RNAs (Claycomb *et al.* 2009, 2009; Corrêa *et al.* 2010), as well as the germ line (Claycomb *et al.* 2009; Boyd *et al.* 2010; Kershner and Kimble 2010; Greer *et al.* 2010; Gracida and Eckmann 2013), epidermal collagens (Rohlfing *et al.* 2010), and neuromuscular lamins (González-Aguilera *et al.* 2014). A KEGG pathway analysis of the genes with negative interactions revealed over-representation of the categories “Base excision repair,” “Homologous recombination,” and “DNA replication,” consistent with germline and early-embryo functions (genes with positive interactions revealed no significant over-represented KEGG categories). Thus, although these analyses revealed enriched functions related to obvious features of phenotypic divergence such as body morphology (Figs. 4b and 5) and developmental timing (Fig. 5), they also revealed surprising sets of genes connected to neuronal, germline, and stress–response roles.

### Genes associated with TGF-β signaling reveal unexpected patterns of transcriptional divergence

**Fig. 6.**
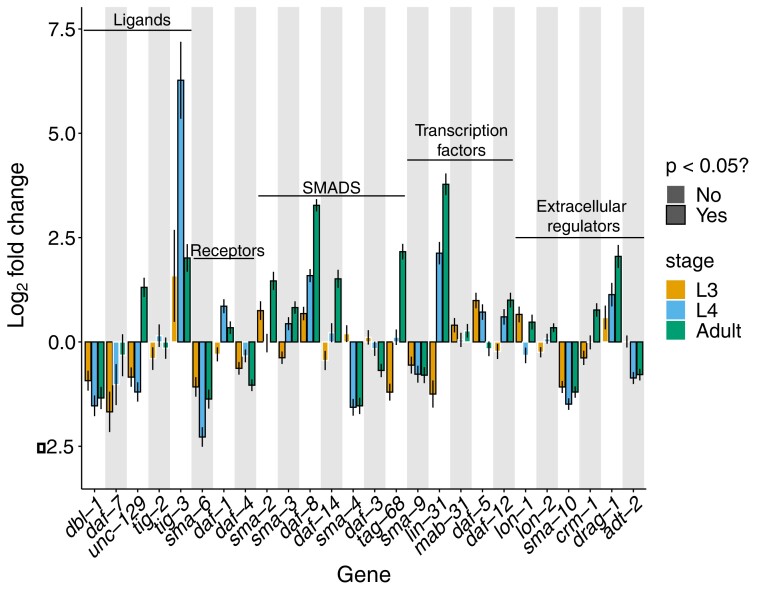
Differential gene expression in genes associated with TGF-β signaling. Plotted are the log_2_ fold changes in transcript abundance of TGF-β signaling-associated genes at each developmental stage between *C. inopinata* and *C. elegans*. Values >0 reveal genes with higher expression in *C. inopinata*, whereas values <0 note genes with higher expression in *C. elegans*. The genes included were extracted from Table 1 of Gumienny and Savage-Dunn 2013. Error bars represent 95% confidence intervals. All *P*-values were corrected for multiple testing (Holm method; see Materials and methods).

In *C. elegans*, body size is regulated by canonical TGF-β signaling (Savage-Dunn and Padgett 2017). Many genes in this pathway impact body size when perturbed (Savage-Dunn and Padgett 2017). For instance, loss-of-function mutations in the extracellular proteins LON-1 (short for “long”; Morita *et al.* 2002) and LON-2 (Gumienny *et al.* 2007) promote increased body size. Additionally, proteins such as the TGF-β ligand DBL-1 (Decapentaplegic/Bone morphogenetic protein-Like) reveal dose-dependent effects on body size—loss-of-function mutants are small whereas overexpression mutants are long (Suzuki *et al.* 1999; Morita *et al.* 1999). Background knowledge regarding the phenotypic effects of mutations in this pathway can thus inform hypotheses regarding the developmental basis of body size evolution. For instance, *C. inopinata* may be long because it has increased DBL-1 expression and/or decreased LON-1 expression. To address these and other possibilities, we examined differential patterns of 26 genes associated with TGF-β signaling that were identified in a previous review (Gumienny and Savage-Dunn 2013) (Fig. 6).

Like most genes, there is extensive differential gene expression between *C. elegans* and *C. inopinata* across TGF-β pathway genes (Fig. 6). However, their differential expression is idiosyncratic (i.e. varied in direction; Fig. 6) and often discordant with the increased body size of *C. inopinata*. For instance, *dbl-1* reveals lower expression in *C. inopinata* at all developmental stages compared to *C. elegans* (Fig. 6), contrary to expectations from the literature (Suzuki *et al.* 1999). Additionally, although *lon-1* and *lon-2* might be expected to have lower expression in the elongated *C. inopinata* (Morita *et al.* 2002; Gumienny *et al.* 2007), the difference in expression compared to *C. elegans* is negligible or even greater (Fig. 6). Yet, a handful of these genes reveal an increase in differential expression across the L4-adult transition (*tig-3*, *daf-8*, *lin-31*, and *drag-1*; Fig. 6), although these genes are not reported to control body size in *C. elegans*.

Patterns of differential expression of TGF-β signaling genes then do not straightforwardly align with the hypothesis that this pathway drives body size evolution in *C. inopinata*. But it is possible that body size mutants in *C. elegans* harbor a transcriptomic signature that is mirrored in *C. inopinata*. To address this, the top decile of genes with significant species–stage interactions (used in gene enrichment analyses; Figs. 4 and 5) were compared with gene lists from previous studies measuring transcriptomic changes in *C. elegans* TGF-β pathway mutants (Liang *et al.* 2007; Roberts *et al.* 2010; Lakdawala *et al.* 2019). The top decile of differentially expressed genes across the L3, L4, and adult stages (Fig. 2) were also compared with the gene lists from these studies. No significant overlap was detected between the most differentially expressed (Fig. 2a–c) or dynamically divergent (Fig. 2d) genes identified here and those associated with *C. elegans* TGF-β pathway mutants (Hypergeometric test Holm-adjusted *P* > 0.05). However, the gene list of one study was dominated by collagen genes (15/18 significant differentially expressed genes encoded collagen proteins) (Lakdawala *et al.* 2019), reminiscent of genes with negative species–stage interactions (Fig. 3b). Thus, while the transcriptome of *C. inopinata* has not diverged in a manner that strictly overlaps patterns seen in TGF-β pathway mutants, many cuticle collagens are differentially expressed in such mutants and exhibit divergent developmental dynamics across species.

### Genes that are not single-copy orthologues also exhibit transcriptional changes across *C. inopinata* development

The above analyses only consider genes that are one-to-one orthologues across *C. elegans* and *C. inopinata*. However, these genes represent only about half of the estimated protein-coding genes in the *C. inopinata* genome (10,718/21,442 or 0.499). Analyses that included all *C. inopinata* coding sequences were also performed to understand the extent of developmental changes in transcript abundance across all genes (Supplementary Figs. 3–7). Much like single-copy orthologues considered in isolation, most genes exhibited transcriptional changes across development (Supplementary Fig. 3; 11,084 genes; LRT Benjamini–Hochberg (BH)-adjusted *P* < 0.05). Genes with a positive transcriptional trajectory over developmental time (8,427) nearly doubled the number of genes whose transcriptional abundance decreased across development (4,426; Supplementary Fig. 3a; Supplementary Tables 8 and 9). Developmentally dynamic genes were also enriched for single-copy orthologues (Supplementary Fig. 3b; χ^2^ BH-adjusted *P* < 0.05; Supplementary Table 12). Additionally, genes with positive developmental trajectories were enriched for protein domains such as major sperm protein, PapD-like, collagens, and tyrosine phosphatases (χ^2^ BH-adjusted *P* < 0.05; Supplementary Table 10). Transcripts with negative developmental trajectories were enriched for protein domains such as collagen and von Willebrand factor (χ^2^ BH-adjusted *P* < 0.05; Supplementary Table 11).

In addition to examining transcriptional abundances over developmental time, we also performed clustering analyses to identify genes harboring similar transcriptional patterns. WGCNA aims to find modules of genes with high interconnectivity in a network framework and uses pairwise correlations among genes to identify such modules (Langfelder and Horvath 2008). Using this approach, 52 modules were identified, with 12 modules harboring significant transcriptional changes over development (Supplementary Fig. 4; linear model BH-adjusted *P* < 0.05; Supplementary Table 16). Most of these modules associated with developmentally dynamic transcription also tended to be enriched for single-copy orthologues (Supplementary Fig. 5; Supplementary Table 15), with the exception of Module 38, which was dominated by multi-copy and *C. inopinata*-specific genes (Supplementary Fig. 5). Module 38 genes tended to have low transcription in the L3 stage, with higher transcription at the L4 and adult stages (Supplementary Fig. 4). Most genes in this module did not have any detected domains, although this module was enriched for chymotrypsin family peptidase domains (Supplementary Table 14). In addition, we also performed hierarchical clustering [using the complete link method (Murtagh and Contreras 2012)] and examined clusters (with *k* = 20; Supplementary Table 17). Here, all but one cluster were found to harbor transcripts with significant developmental dynamics (Supplementary Table 18; Supplementary Fig. 6–7; linear model BH-adjusted *P* < 0.05). Here, clusters with high positive trajectories were enriched for genes in multi-copy gene families and *C. inopinata*-specific genes (Supplementary Fig. 7; χ^2^ BH-adjusted *P* < 0.05; Supplementary Table 20). Conversely, clusters with steep negative trajectories were enriched for single-copy orthologues (Supplementary Fig. 7; χ^2^ BH-adjusted *P* < 0.05; Supplementary Table 20). One cluster was notable for harboring transcripts with high expression at the L4 stage specifically (Cluster 19, Supplementary Fig. 7a); this cluster was also enriched for genes in multi-copy gene families and *C. inopinata*-specific genes (Supplementary Fig. 7b; χ^2^ BH-adjusted *P* < 0.05; Supplementary Table 20). This cluster harboring genes with high L4 expression was enriched for genes with collagen domains (Supplementary Table 19). Clusters with positive developmental trajectories were enriched for domains related to major sperm protein (Cluster 8), chitin (Cluster 15), collagen (Cluster 15 and 20), and vitellogenin (Cluster 20; Supplementary Table 19). Clusters with negative developmental trajectories were enriched for collagen domains (Cluster 16 and 17). These results then bear some similarities to those with single-copy orthologues alone, with collagen and reproductive genes harboring dynamic transcriptional profiles across the larval-to-adult transition.

### Genes that align to transposons exhibit lower expression

**Fig. 7.**
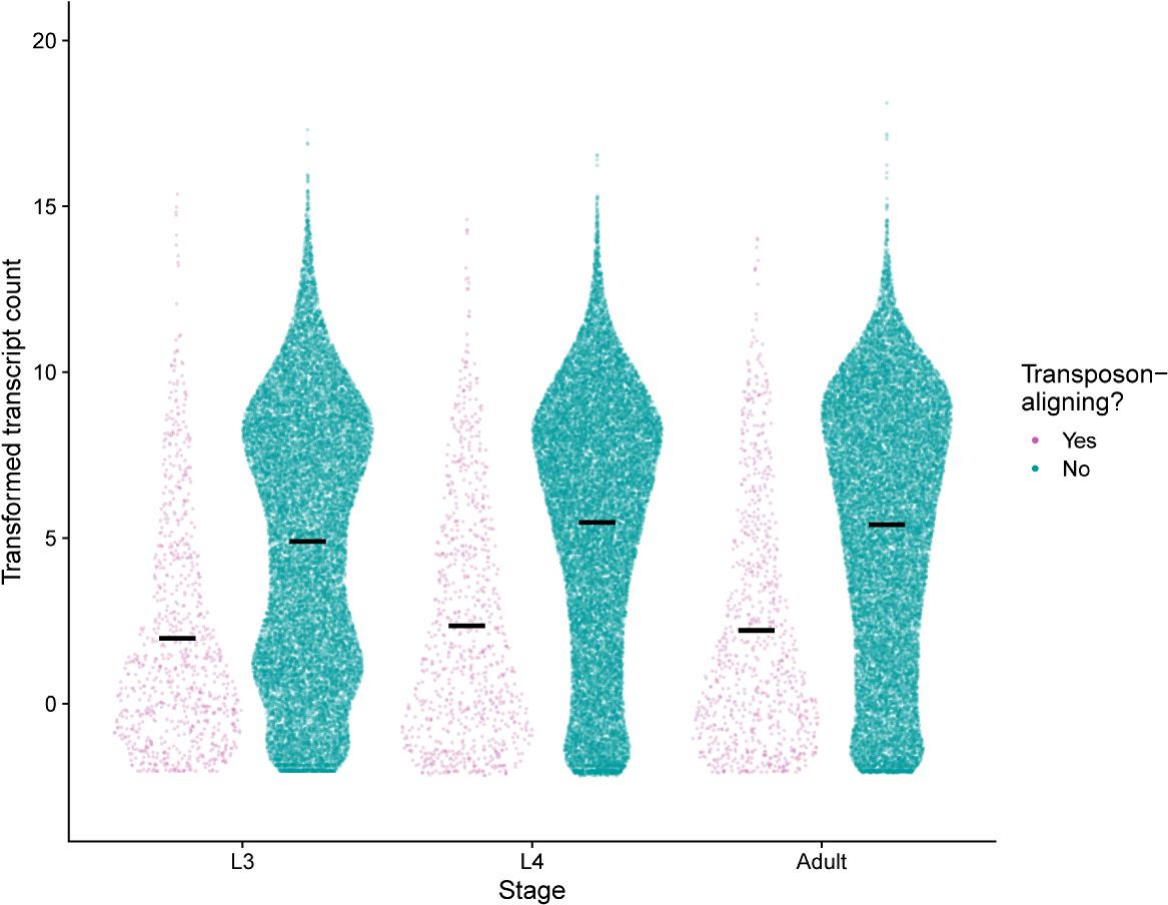
Transposon-related genes exhibit lower expression in *C. inopinata*. Plotted are the transcript counts (regularized log-transformed) of *C. inopinata* genes whose encoded proteins either align with transposon-related proteins (originally identified in Woodruff and Teterina 2020) or not. Sina plots are strip charts with points taking the contours of a violin plot. Black horizontal bars represent means.

*C. inopinata* does not only harbor a unique body size and morphology—it also harbors an unusual repetitive genomic landscape (Kanzaki *et al.* 2018; Woodruff and Teterina 2020). *C. inopinata* maintains many open reading frames (ORFs) encoding proteins related to transposable elements (Woodruff and Teterina 2020). To understand the biological activity of these genes, we compared transcriptional abundances of these transposon-aligning genes with those that do not align to transposons (Fig. 7). Across all developmental stages observed, transposon-aligning genes harbor far lower expression than genes that do no align to transposons (Fig. 7; 57–60% reduction in transformed transcript count; Cohen's *d* effect size = −0.82 to −0.73; Wilcoxon rank-sum test *P* < 0.001). Despite this, there are transposon-aligning genes with high transcriptional abundance (Fig. 7), suggesting these ORFs maintain some degree of biological activity.

### Species-specific amino acid replacements are common and correlate with phylogenetic distance

Transcriptional patterns of divergence are being measured to generate hypotheses regarding the genetic bases of body size evolution. However, changes in protein-coding genes are also expected to potentially promote phenotypic evolution. To address this, we also looked at species-specific amino acid replacements across 14 species of the *Elegans* group of *Caenorhabditis* (*C. kamaaina*, *C. inopinata*, *C. elegans*, *C. brenneri*, *C. doughertyi*, *C. tropicalis*, *C. wallacei*, *C. latens*, *C. remanei*, *C. briggsae*, *C. nigoni*, *C. sinica*, *C. zanzibari*, and *C. tribulationis*). Trimmed alignments of 2,767 single-copy protein orthologues across all of these species were generated (including only those alignments >19 amino acids in length). Species-specific amino acid replacements for all species were then identified and counted. On average, 1% of a protein's amino acids are represented by species-specific amino acid replacements [Supplementary Fig. 8; 0.0083 mean species-specific replacement fraction (species-specific amino acid replacements)/(total amino acids in given protein)]. Including all proteins and all species, there was an average of 7,513 species-specific replacements per species. However, species that are more phylogenetically divergent tend to have more species-specific replacements (Supplementary Figs. 8 and 9). For instance, *C. remanei*, which has only recently diverged from *C. latens* (Dey *et al.* 2012; Félix *et al.* 2014; Fierst *et al.* 2015; Teterina *et al.* 2023), has an average of 0.0028 species-specific amino acids per total protein length (1,804 species-specific replacements), whereas the early-diverging *C. kamaaina* has a mean value of 0.023 (21,148 species-specific amino acid replacements). Indeed, there is a strong correlation between terminal phylogenetic branch length and mean species-specific replacement fraction (β = 0.17; *r^2^* = 0.94; *P* < 0.001; Supplementary Fig. 9), and *C. elegans* has a nontrivial fraction of species-specific replacements per protein (0.013; 12,221 total species-specific amino acid replacements). Likewise, *C. inopinata* harbors a long terminal branch and many species-specific replacements (0.027 species-specific replacements/total amino acids; 24,691 total species-specific amino acid replacements). Despite their correlation with phylogenetic distance, we isolated proteins with a high fraction of species-specific amino acid replacements in *C. inopinata* [>3 times one standard deviation of the mean (including all species) or >0.057]. This yielded 233 proteins (Supplementary Table 18) that were analyzed with the WormBase gene set enrichment tool. Proteins with high numbers of species-specific replacements in *C. inopinata* were enriched for excretory duct and excretory socket cell expression (WormBase Anatomy Ontology; Supplementary Table 22). They were likewise enriched for hyperactive foraging phenotypes (WormBase Phenotype Ontology; Supplementary Table 22). Additionally, these genes were enriched for neuropeptide signaling, cuticle development, molting cycle, and lipid metabolism processes (Gene Ontology; Supplementary Table 22).

## Discussion

### Widespread transcriptional divergence across postembryonic development

Over half of about 11,000 single-copy orthologues are differentially expressed between *C. elegans* and *C. inopinata* at all developmental stages considered. This is surprising because these species are closely related and harbor similar developmental patterns (Woodruff *et al.* 2018; Kanzaki *et al.* 2018). No differences in the number of somatic cells between *C. elegans* and *C. inopinata* adults could be detected in a previous study (Woodruff *et al.* 2018), suggestive of a highly conserved cell lineage among the 2 species. Thus, we might potentially expect highly conserved patterns of gene expression to co-occur with these developmental similarities. One potential explanation for these differences could be the use of a *fog-2* mutation in our *C. elegans* populations; this is an unlikely driver of apparent rampant gene expression divergence because this gene's function appears to be limited to hermaphrodite germline sex determination and has no other clear impacts on fitness (Schedl and Kimble 1988). A more likely explanation for the expression divergence is the morphological and ecological divergence among these species. Not only is *C. inopinata* much longer than *C. elegan*s—it also thrives in a markedly different natural environment (fresh *F. septica* figs instead of rotting plants) (Kanzaki *et al.* 2018; Woodruff and Phillips 2018). Its radically divergent transcriptome may then reflect its divergent morphology and ecology, and *C. inopinata* in particular may require the ubiquitous tuning of gene expression to shape its needs as a fig nematode.

Another possible explanation is that such divergent patterns of gene expression are common among closely related nematode species. In other words, developmental system drift may explain these differing transcriptomic dynamics (True and Haag 2001). The extent of differential gene expression across postembryonic development in *Caenorhabditis* nematodes is not entirely obvious. Unexpectedly divergent expression across embryogenesis has been reported in *C. elegans* and *C. briggsae* (Yanai and Hunter 2009). Alternatively, it has been reported that gene expression across postembryonic development is largely conserved between *C. elegans* and *C. briggsae* (Grün *et al.* 2014; Lu *et al.* 2020). It has also been shown that transcriptional patterns are more likely to be conserved during ventral enclosure when compared to other embryonic stages (Levin *et al.* 2012). Additionally, hermaphroditic species tend to have less complex and less sex-biased transcriptomes than gonochoristic species (when considering adults) (Thomas *et al.* 2012), and a transgenic reporter construct survey with 8 genes and 4 *Caenorhabditis* species revealed widespread spatial variation in gene expression (Barrière and Ruvinsky 2014). Notably, over half of the genes examined were found to be differentially expressed between *C. briggsae* and *C. nigoni* nematodes of the same sex (Sánchez-Ramírez *et al.* 2021). As these species are far more closely related to each other than *C. inopinata* and *C. elegans* (Sloat *et al.* 2022), this lends support to the view that developmental system drift in transcriptomes is common in this group. Regardless, future studies that capture a larger phylogenetic sample as well as a range of postembryonic stages will be required to disentangle these possibilities.

Additionally, it is important to address some caveats regarding the comparison of nematode transcriptomes among species. Here, nematode populations were pooled and used for RNA extractions. This has the potential to lead to some biases that may influence our results. For instance, we used mixed-sex populations. If there are species-specific biases in sex ratios among our groups, this may lead to the erroneous inference of widespread transcriptional divergence among species. Indeed, sex ratio biases in *Caenorhabditis* populations have been observed (Huang *et al.* 2023), although this has not been clearly seen in *C. elegans* (Hodgkin *et al.* 1979; Teotónio *et al.* 2006; Huang *et al.* 2023) or *C. inopinata* (Woodruff *et al.* 2019). Additionally, nematode population extracts might lead to tissue-specific biases—if certain tissues vary in their propensity to be degraded among groups, this may underlie inferences of interspecific transcriptional change. One potential driver of this is the known differences in germline size in *C. inopinata* compared to *C. elegans*; *C. inopinata* appears to have much smaller gonads than *C. elegans* (Woodruff *et al.* 2018), despite its elongated body size. Further complicating transcriptomic comparisons of *C. elegans* and *C. inopinata* are known differences in life-history traits under laboratory conditions (Woodruff *et al.* 2019). Specifically, *C. inopinata* has lower fecundity, slower rates of development, and lower viability than *C. elegans* in such environments (Woodruff *et al.* 2019). It is then possible that different rearing conditions may promote a better alignment of transcriptional patterns among species. That is, this seemingly widespread transcriptional divergence may be driven by variation in optimal environmental conditions rather than by any intrinsic differences related to morphological or reproductive divergence. Regardless, further studies examining sex-specific, tissue- (and cell-) specific, and context-specific transcription will be needed to reveal if such biases impact these findings.

### Transcriptional divergence and body size evolution

We were able to identify transcriptional patterns of many orthologues connected to TGF-β signaling. This pathway influences body size in *C. elegans*, and numerous mutants associated with this pathway exhibit small or elongated bodies. Surprisingly, our results revealed idiosyncratic patterns of differential gene expression that do not align with simple models of TGF-β signaling (Fig. 6). For instance, in *C. elegans*, high levels of *dbl-1* transcription promote repression of the downstream target gene *lon-1*, leading to body size increases (Morita *et al.* 2002). Thus, a natural hypothesis would be that *C. inopinata* is long due to increased *dbl-1* (and decreased *lon-1*) expression. Neither of these patterns was observed; *dbl-1* is *lower* in expression while *lon-1* exhibits negligible differences in gene expression (Fig. 6). Thus, it is unlikely that body size is driven by transcriptional evolution of *dbl-1*, *lon-1*, or other TGF-β signaling genes in a manner concordant with such simple hypotheses derived from *C. elegans* developmental genetics. This is consistent with the observation that *C. inopinata* does not harbor differences in hypodermal endoreplication compared to *C. elegans* (Woodruff *et al.* 2018); TGF-β signaling has been proposed to regulate body size through this mechanism (Lozano *et al.* 2006). Additionally, Sma/Mab TGF-β signaling components appear to regulate body size during the early larval stages of *C. elegans* development (Savage-Dunn *et al.* 2000; Liang *et al.* 2003). As much of the body size difference in *C. inopinata* is established during the larval-to-adult transition, this would also suggest that TGF-β signaling may not be implicated in its elongated body size (Woodruff *et al.* 2018). However, gene functions have been shown to evolve in *Caenorhabditis* nematodes (Beadell *et al.* 2011; Verster *et al.* 2014), and it is entirely possible that the roles of TGF-β pathways have changed in *C. inopinata* (which can then resolve these unexpected transcriptional patterns). Future studies involving the perturbation of these genes’ activities in *C. inopinata* will be required to interrogate this possibility.

Notably, cuticle collagens were common among genes with negative species–stage interactions (Fig. 3). *C. elegans* harbors over a hundred such genes (Teuscher *et al.* 2019), and a number of genes with morphological mutant phenotypes encode such collagens (Johnstone 2000) (although most collagen genes have no described phenotypes). As these genes encode core components of the extracellular matrix constituting the exoskeleton, it is unsurprising that some of these genes regulate body morphology. Moreover, collagen genes have been shown to be regulated by TGF-β signaling (Madaan *et al.* 2018, 2020), and this pathway may regulate body morphology in part by controlling the expression of such exoskeletal factors. It is then possible that these divergent transcriptional dynamics in collagen genes may promote the evolution of elongated body size in *C. inopinata*.

Additionally, as single-copy orthologues represent only about half of the protein-coding genes of *C. inopinata*, we also examined transcriptional patterns in *C. inopinata* irrespective of their relationship with *C. elegans*. Indeed, a majority of genes harbor transcriptional relationships with developmental time (Supplementary Fig. 3). Like the single-copy orthologues, genes with both positive and negative transcriptional trajectories over development include collagens and reproduction-related genes (such as those with major sperm protein domains; Supplementary Tables 10 and 11). We also identified clusters of genes bearing similar patterns of transcriptional dynamics across postembryonic development (Supplementary Figs. 4–7). These clusters also included many genes that do not have clear single-copy orthologues in *C. elegans* (Supplementary Figs. 6 and 7b). Thus, patterns of gene duplication, gene loss, and new gene origination (in addition to transcriptional divergence among single-copy orthologues) are also likely to contribute to the evolution of body elongation in *C. inopinata*.

### Stress, behavior, small RNAs, and the germ line

Enrichment analysis of divergently dynamic genes generated results concordant with *C. inopinata*'s divergent body size (“body morphology variant” and “dumpy” WormBase Phenotypes; Fig. 4) and developmental rate (“expression oscillatory during larval development” WormExp experiment (Hendriks *et al.* 2014; Fig. 5). However, enrichment analyses also detected a range of unexpected biological phenomena associated with divergent developmental transcriptional dynamics. For instance, overlap was found between divergently dynamic genes and genes exhibiting differential expression in a number of experiments related to stress–response [including genes such as *daf-2* (Zarse *et al.* 2012), *daf-16* (Delaney *et al.* 2017), and *daf-12* (Delaney *et al.* 2017); Fig. 5]. Additionally, genes connected to neurons (Fig. 4a) and behavioral phenotypes (Fig. 4b) were also enriched. Some of these behavioral phenotypes (such as “copulation” and “male mating efficiency”) are likely due to the difference in reproductive mode between species. *C. elegans* is a self-fertile hermaphrodite harboring low male frequencies in natural populations (Cutter *et al.* 2019); *C. inopinata* is an obligate outcrosser (Kanzaki *et al.* 2018). However, we speculate these other differences result from these species” divergent natural ecological contexts. *C. elegans* thrives in rotting plant material (Frézal and Félix 2015) and grows readily in laboratory conditions. *C. inopinata* thrives in fresh figs (Kanzaki *et al.* 2018; Woodruff and Phillips 2018) and has low fecundity in laboratory conditions (Woodruff *et al.* 2019). Thus, its behavioral and stress-response regimes are likely to be tuned to a radically different natural context, thus driving patterns of divergently developmentally dynamic expression in the genes underlying these biological functions. Consistent with this, the stress-resistant dauer stage has diverged in *C. inopinata*, exhibiting an apparent loss of radial constriction and a far lower prevalence in laboratory conditions than *C. elegans* (Hammerschmith *et al.* 2022). Thus, while it is possible there may be some co-option of behavioral and stress genes in the divergent growth and developmental processes of *C. inopinata*, it is more likely that these traits themselves have diverged in this lineage.

Germline genes were also detected in both enrichment analyses performed (Figs. 4c, d and 5b). This is also likely due to *C. inopinata*'s divergent environmental context and low fecundity in laboratory conditions. A previous study revealed the adult female gonad of *C. inopinata* is much smaller and holds far fewer germ cells than that of *C. elegans* (Woodruff *et al.* 2018). Indeed, if somatic and germ cells are included, *C. elegans* has more cells than *C. inopinata* despite its smaller body size (Woodruff *et al.* 2018). Additionally, these enrichment analyses were performed on samples undergoing maturation—if patterns of oogenesis and early embryogenesis are divergent [which has been observed in *Caenorhabditis* (Yanai and Hunter 2009; Levin *et al.* 2012; Farhadifar *et al.* 2015)], then it would be unsurprising to see such divergent dynamics of germline genes in our samples. Connected to this, genes associated with small RNA biology were also detected in enrichment analyses [such as *csr-1* (Claycomb *et al.* 2009), *alg-1* (Zisoulis *et al.* 2010), and *mir-243* (Corrêa *et al.* 2010); Fig. 5). This may simply reflect the potential germ line divergence described above. The germ line harbors an array of tissue-specific granules that contain small RNAs (Sundby *et al.* 2021), and germ line divergence may entail the evolution of germ granules. However, small RNAs (particularly piRNAs) are known to regulate transposable elements (Tóth *et al.* 2016), and the genome of *C. inopinata* has evolved a highly repetitive (Kanzaki *et al.* 2018) and surprisingly uniform (Woodruff and Teterina 2020) landscape of such elements. Moreover, *C. inopinata* has lost the key small RNA regulators *ergo-1*, *eri-9*, and *eri-6/7* (Kanzaki *et al.* 2018). The divergent dynamics of small RNA genes may also then be connected to *C. inopinata*'s exceptionally transposable element-rich genome and the loss of conserved small RNA machinery.

### Transposable elements

In addition to having a transposon-rich genome in general, *C. inopinata* has many ORFs that encode transposon-related proteins (such as integrases, polymerases, ribonucleases, etc.) (Woodruff and Teterina 2020). Transposons are expected to be deleterious to the host (Wicker *et al.* 2007), and as a consequence, myriad defenses have evolved to silence such elements and inhibit their activity (Buchon and Vaury 2006; Tóth *et al.* 2016). Here, we showed that these transposon-aligning ORFs reveal a 50% reduction in mean transformed transcript count compared to genes that do not align to transposons (Fig. 7). This is consistent with these ORFs being deleterious and with host inhibition of their activity. However, many of these genes are highly expressed (Fig. 7). This suggests that many of these genes are active transposable elements or otherwise harbor activity that is biologically relevant to the host. Disentangling these possibilities will require whole-genome sequencing of either multiple *C. inopinata* populations [which has been reported to be in progress (Kawahara *et al.* 2023)] or longitudinal studies of single populations to track the transposition of active elements. Indeed, a recent study showed that *C. inopinata*-specific transposable element insertions are associated with changes in gene expression across species (Kawahara *et al.* 2023). And, ancient horizontal transfer of transposable elements has been suggested to drive reproductive isolation in *Caenorhabditis* nematodes (Widen *et al.* 2023). Beyond this, it remains possible that transposon-associated ORFs have been co-opted for host functions (Jangam *et al.* 2017; Singh and Bhalla 2020), and future studies will be required to address this possibility.

### Species-specific amino acid replacements

Transcriptional change is not the only consequence of divergence. Protein sequences themselves also evolve, and such changes can have profound phenotypic impacts. A longstanding question within evolutionary developmental biology is the relative importance of regulatory sequence change compared to protein-coding change with respect to morphological evolution (Hoekstra and Coyne 2007; Stern and Orgogozo 2008). While our study focused on changes in gene expression, other genomic changes are likely to be critical for body size evolution. To this end, we also examined the extent of species-specific amino acid replacements across 2,767 single-copy orthologous proteins among fourteen *Caenorhabditis* species (Supplementary Fig. 8). All species harbor species-specific amino acid replacements, but *C. inopinata* harbors the most out of all species considered (Supplementary Fig. 8). Thus, there are a great number of potential substitutions that may be relevant for the evolution of body size in this species. However, *C. elegans* also has a high number of species-specific amino acid replacements (Supplementary Fig. 8). As these values are correlated with phylogenetic distance (Supplementary Fig. 9), it is not necessarily surprising that this widely studied model organism is one harboring a large number of apparent species-specific variants. That is, neutral amino acid replacements are expected to accumulate over evolutionary time (Kimura 1987). Thus, while such replacements can be counted, it is difficult to predict their phenotypic relevance. Additionally, it is important to note that this analysis did not take into account intraspecific variation, and it is entirely possible that many of these apparent species-specific variants actually represent sites harboring within species polymorphisms. Regardless, we identified 233 proteins with high numbers of species-specific amino acid replacements in *C. inopinata*, and these were enriched for processes related to foraging, the excretory duct, and lipid metabolism, among others (Supplementary Tables 21 and 22). As *C. inopinata* reveals numerous divergent phenotypes compared to its close relatives (Kanzaki *et al.* 2018; Woodruff and Phillips 2018; Woodruff *et al.* 2018; Woodruff *et al.* 2019; Woodruff and Teterina 2020; Hammerschmith *et al.* 2022), it is possible that the evolution of these proteins have contributed to changes in behavior, metabolism, and the excretory system. Phylogenetic comparative methods using mRNA alignments may prove useful in discovering evolutionarily relevant amino acid replacements. Regardless, future investigations examining the extent of divergence in these phenotypes, as well as the relevance of these amino acid replacements to these kinds of phenotypic changes, will be required to inform these possibilities.

### Findings in light of Kawahara *et al. GBE* 2023

Recently, a study performing a very similar set of experiments was published (Kawahara *et al.* 2023). How do our results compare? For instance, under principal components analysis, the first 2 principal components of both studies separate samples of differing stages and species (compare Supplementary Fig. 1 of Kawahara *et al.* 2023 with Supplementary Fig. 1 of this study). Moreover, they found that 64–71% of single-copy orthologues were differentially expressed across species, even greater than our observations (55–66%). Thus, both studies suggest body size evolution is accompanied by widespread gene expression divergence. Additionally, Kawahara *et al.* reported notable divergence in collagen gene expression, as well as idiosyncratic expression across the TGF-β signaling pathway, consistent with our findings. Additionally, their detailed analyses of transposable element insertion impacts on orthologue expression are consistent with our findings that transposon-aligning genes are expressed. Thus, our second report confirms these broad conclusions are robust. That said, there are more specific, quantitative differences between these reports. For instance, Kawahara *et al.* detected no differential expression in *dbl-1* (their Supplementary Fig. 6), whereas we found this gene is under-expressed in *C. inopinata* at all developmental stages (Fig. 6). While it is unclear exactly what is driving these discordant findings, it is worth noting there were a number of biological differences among the studies that may explain specific differences in transcript abundances. For instance, in our study, all animals were grown at 25°C on *E. coli*OP50-1. In Kawahara *et al.* 2023, *C. inopinata* was grown on *E. coli* strain HT115 (DE3) at 27°C, while *C. elegans* was grown at 24.5°C. Additionally, we used *C. elegans fog-2 (q71)* to account for reproductive mode, whereas Kawahara *et al.* 2023 used *fem-3 (hc17)*. Indeed, our populations included males, whereas Kawahara *et al.* 2023 only examined females. Thus, the environments, sexual compositions, and genetic backgrounds differed across these studies; this is likely to explain such specific differences in transcription. It is thus all the more striking that the broad conclusions of these studies are robust to such differences in experimental design.

## Concluding thoughts

Here, we found widespread transcriptional divergence across the larval-to-adult transition between *C. elegans* and *C. inopinata*. While the extent of developmental system drifts in transcript abundance in this group is uncertain, some fraction of these transcriptionally divergent genes must be implicated in the evolution of increased body length in *C. inopinata*. Genes with divergent dynamics included those encoding collagens, those with body morphology size phenotypes in *C. elegans*, and those connected to TGF-β signaling. This work then reveals multiple specific hypothetical drivers of body size in this group and sets the stage for future laboratory experiments interrogating the developmental and genetic bases of body size evolution.

## Supplementary Material

jkae110_Supplementary_Data

## Data Availability

FASTQ files have been submitted to the NCBI Sequence Read Archive (SRA; http://www.ncbi.nlm.nih.gov/sra) under the BioProject ID PRJNA1031217. Sample metadata can be found in supplemental_tables.xls Sheet 1. All other data and code affiliated with this work have been deposited in Github (https://github.com/gcwoodruff/inopinata_developmental_transcriptomics_2023). Supplementary material is available at G3 online.
