# Supplementary material for: Widespread changes in gene expression accompany body size evolution in nematodes
Source: G3 (Bethesda). 2024 May 22;14(8):jkae110. doi: 10.1093/g3journal/jkae110 (PMC11304970; doi:10.1093/g3journal/jkae110)
Supplement: jkae110_Supplementary_Data [file jkae110_supplementary_data.zip › Supplemental_Figures_G3-2024-405083.pdf]

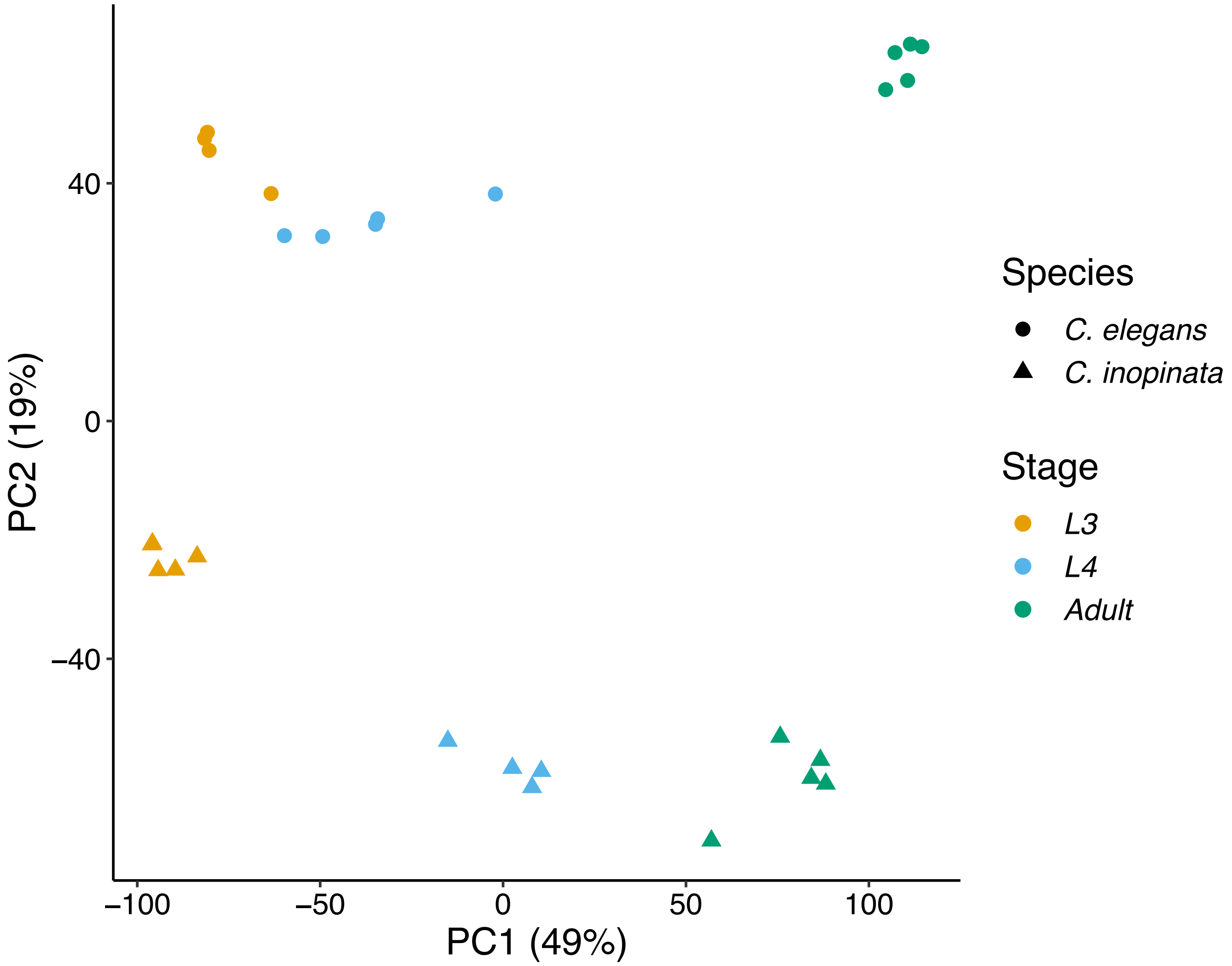

Supplemental figure 1. Principal Component Analysis of all samples. The first two PC’s are plotted.

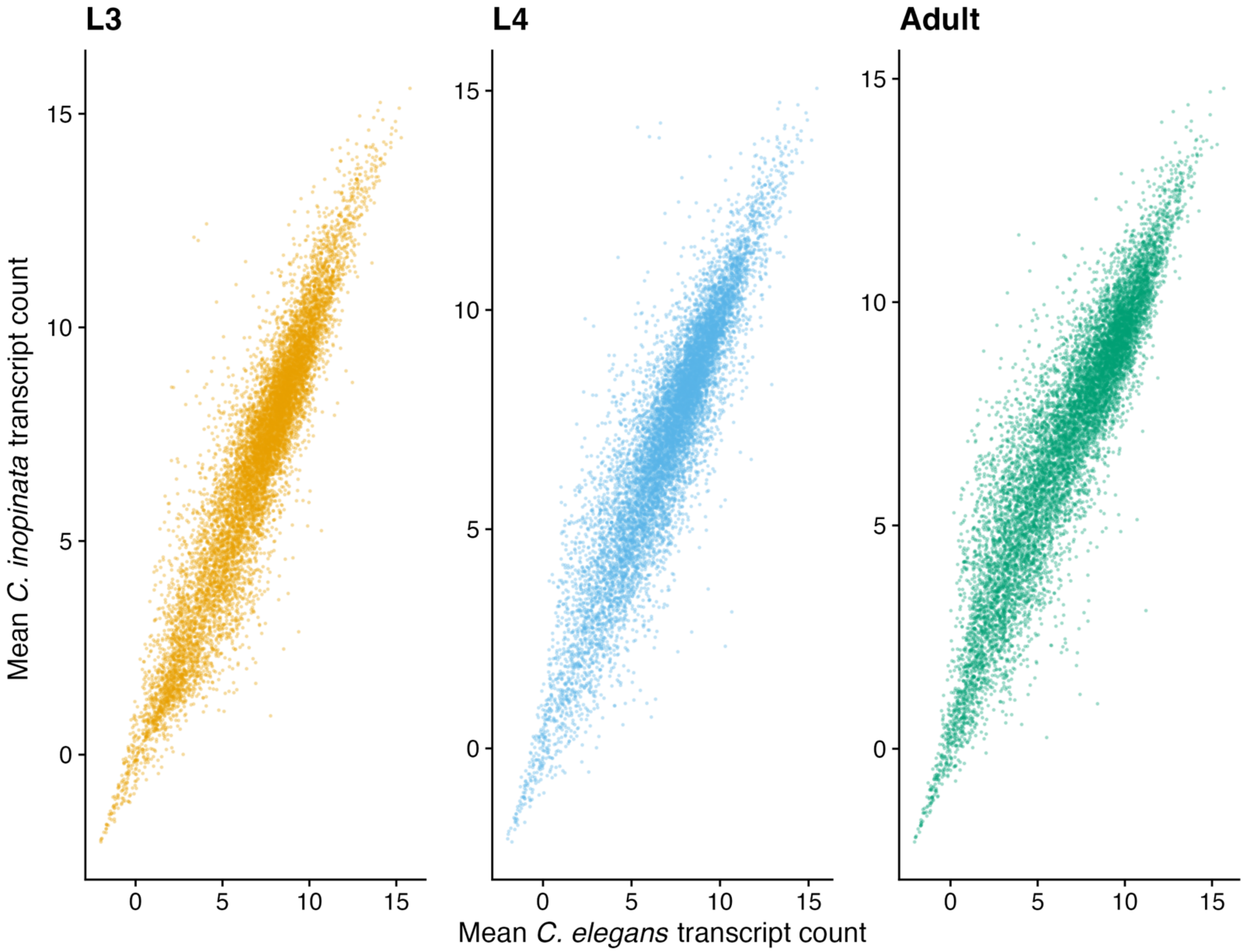

Supplemental figure 2. Comparison of *C. elegans* and *C. inopinata* gene expression at each developmental stage. (A) L3 (B) L4 (C) adult. Plotted are the transcript counts (regularized log transformed) of 10,717 single-copy orthologous genes.

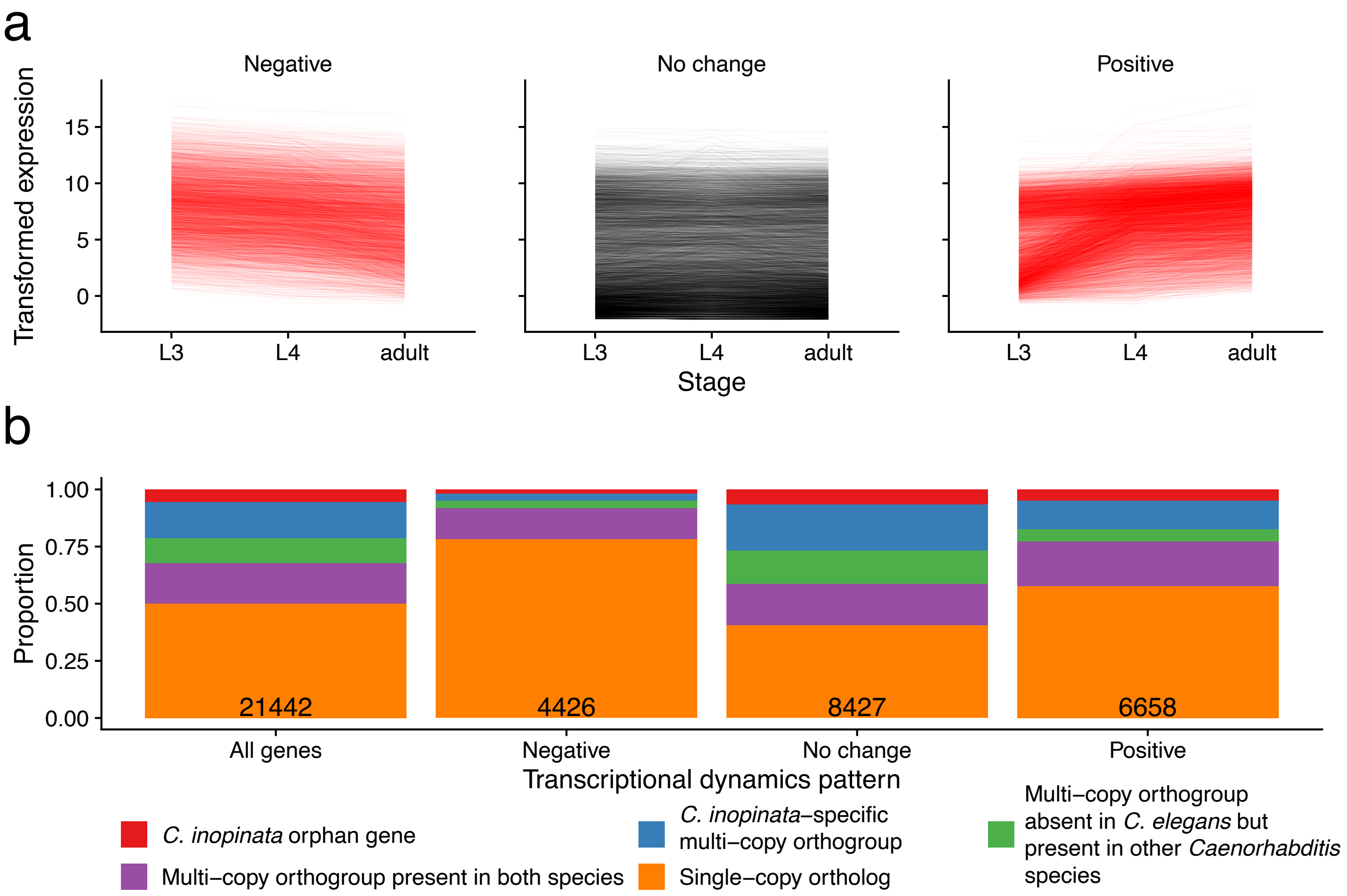

Supplemental figure 3. Developmental dynamics of all *C. inopinata* genes, including those that are not single-copy orthologs. (A) Developmental dynamics of genes binned by negative ($\beta_1 < 0$ and LRT BH-adjusted $p < 0.05$) and positive ($\beta_1 > 0$ and LRT BH-adjusted $p < 0.05$) trajectories. Genes with no significant correlation with developmental time (“No change”, LRT BH-adjusted $p < 0.05$) are also plotted. All *p*-values were adjusted for multiple testing with the BH method (Benjamini and Hochberg 1995; using the *results()* function in the *DESeq2* package). Each line represents the average regularized-log transcript abundance for each gene at each stage in *C. inopinata*. Genes with significant correlations among transcript abundance and developmental time (LRT BH-adjusted $p < 0.05$) are colored red. (B) *C. inopinata* genes classified by orthologous group categories across transcriptional dynamics patterns (positive, negative, or no change, as in panel (A)). The numbers of genes in each transcriptional dynamics pattern are reported at the base of each bar in the plot. Orthogroups were inferred with OrthoFinder among five *Caenorhabditis* species (*C. elegans*, *C. inopinata*, *C. remanei*, *C. briggsae*, and *C. nigoni*). Genes are categorized by the distribution of gene counts across species in the orthologous group to which the given gene belongs:

- *C. inopinata* orphan gene: a *C. inopinata* gene that clusters with *no* other genes.
- *C. inopinata*-specific multi-copy: a *C. inopinata* gene in an orthogroup containing only *C. inopinata* genes.
- Multi-copy orthogroup absent in *C. elegans* but present in other *Caenorhabditis* species: a *C. inopinata* gene in an orthogroup harboring copies in other *Caenorhabditis* species aside from *C. elegans*.
- Multi-copy orthogroup present in both species: a *C. inopinata* gene in an orthogroup harboring more than one gene copy in either *or* both of *C. inopinata* or *C. elegans*.
- Single-copy ortholog: a *C. inopinata* gene in an orthogroup harboring one gene copy in all five *Caenorhabditis* species examined.

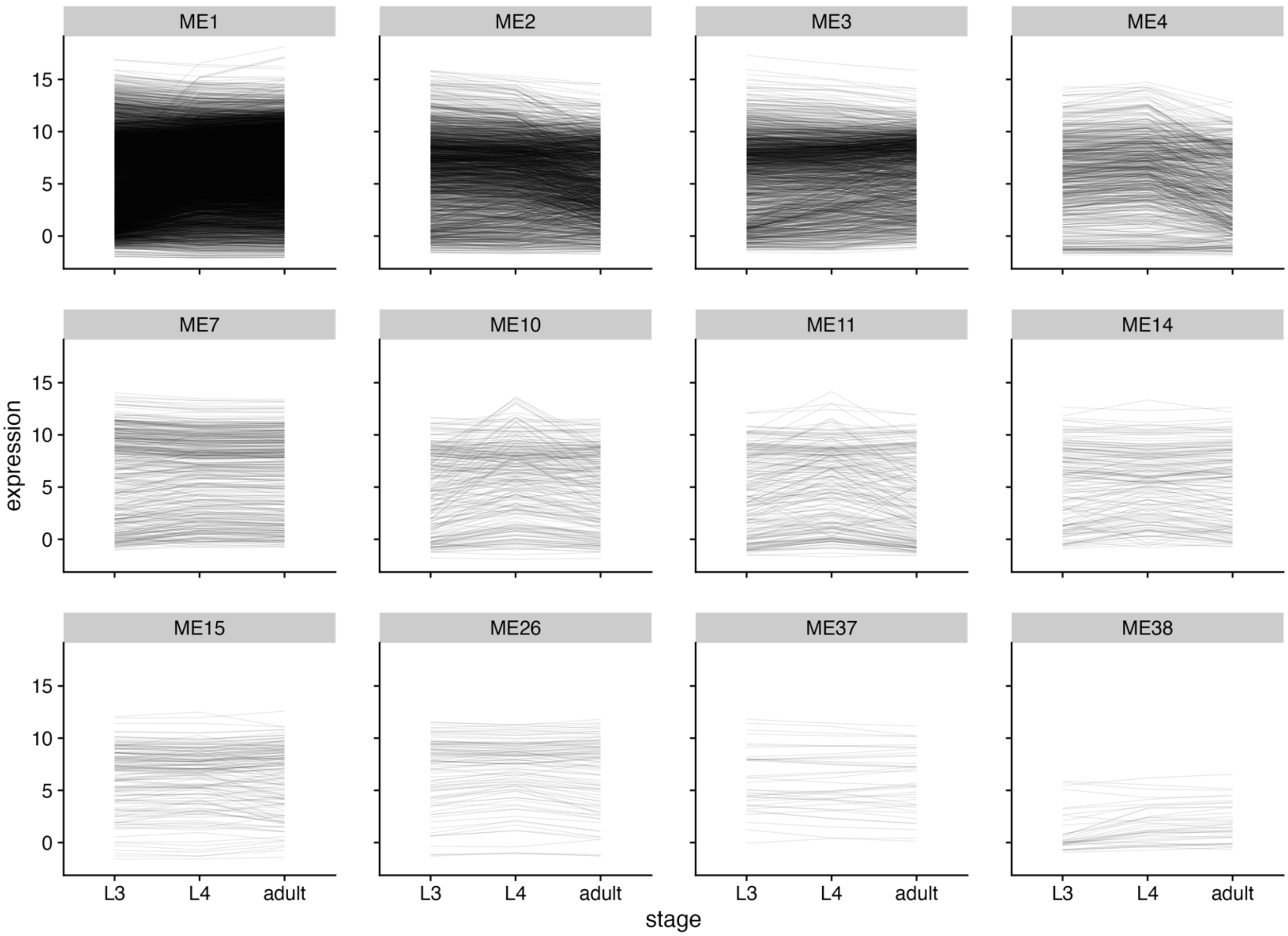

Supplemental Figure 4. WGCNA significant modules using all *C. inopinata* genes. Following WGCNA, linear models were fit to all 52 modules (using the *limma* package *lmFit()* function with default parameters; the model formula "~ Stage" was used). Twelve modules were found to have a significant relationship with developmental stage (BH-corrected F-test $p < 0.05$), and these modules are plotted here. For a given module, each line represents the average regularized-log transcript abundance for each gene at each stage in *C. inopinata*.

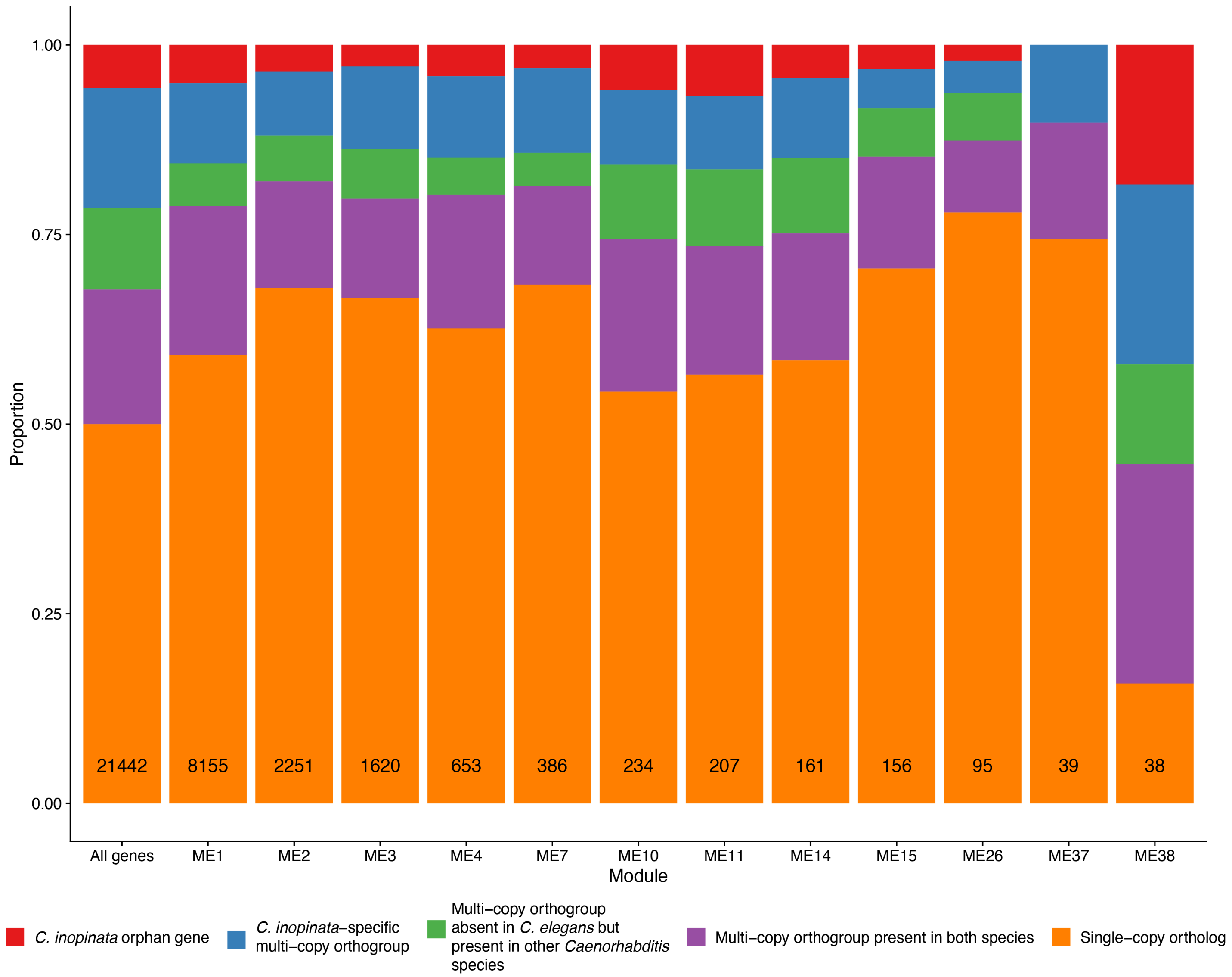

Supplemental Figure 5. Significant WGCNA modules classified by orthologous group categories. Following WGCNA, linear models were fit to all 52 modules (using the *limma* package *lmFit()* function with default parameters; the model formula "~ Stage" was used). Twelve modules were found to have a significant relationship with developmental stage (BH-corrected F-test $p < 0.05$), and these modules are plotted here. The number of genes in each module are reported at the base of each bar in the plot. Orthogroups were inferred with OrthoFinder among five *Caenorhabditis* species (*C. elegans*, *C. inopinata*, *C. remanei*, *C. briggsae*, and *C. nigoni*). Genes are categorized by the distribution of gene counts across species in the orthologous group to which the given gene belongs:

- *C. inopinata* orphan gene: a *C. inopinata* gene that clusters with *no* other genes.
- *C. inopinata*-specific multi-copy: a *C. inopinata* gene in an orthogroup containing only *C. inopinata* genes.
- Multi-copy orthogroup absent in *C. elegans* but present in other *Caenorhabditis* species: a *C. inopinata* gene in an orthogroup harboring copies in other *Caenorhabditis* species aside from *C. elegans*.
- Multi-copy orthogroup present in both species: a *C. inopinata* gene in an orthogroup harboring more than one gene copy in either *or* both of *C. inopinata* or *C. elegans*.
- Single-copy ortholog: a *C. inopinata* gene in an orthogroup harboring one gene copy in all five *Caenorhabditis* species examined.

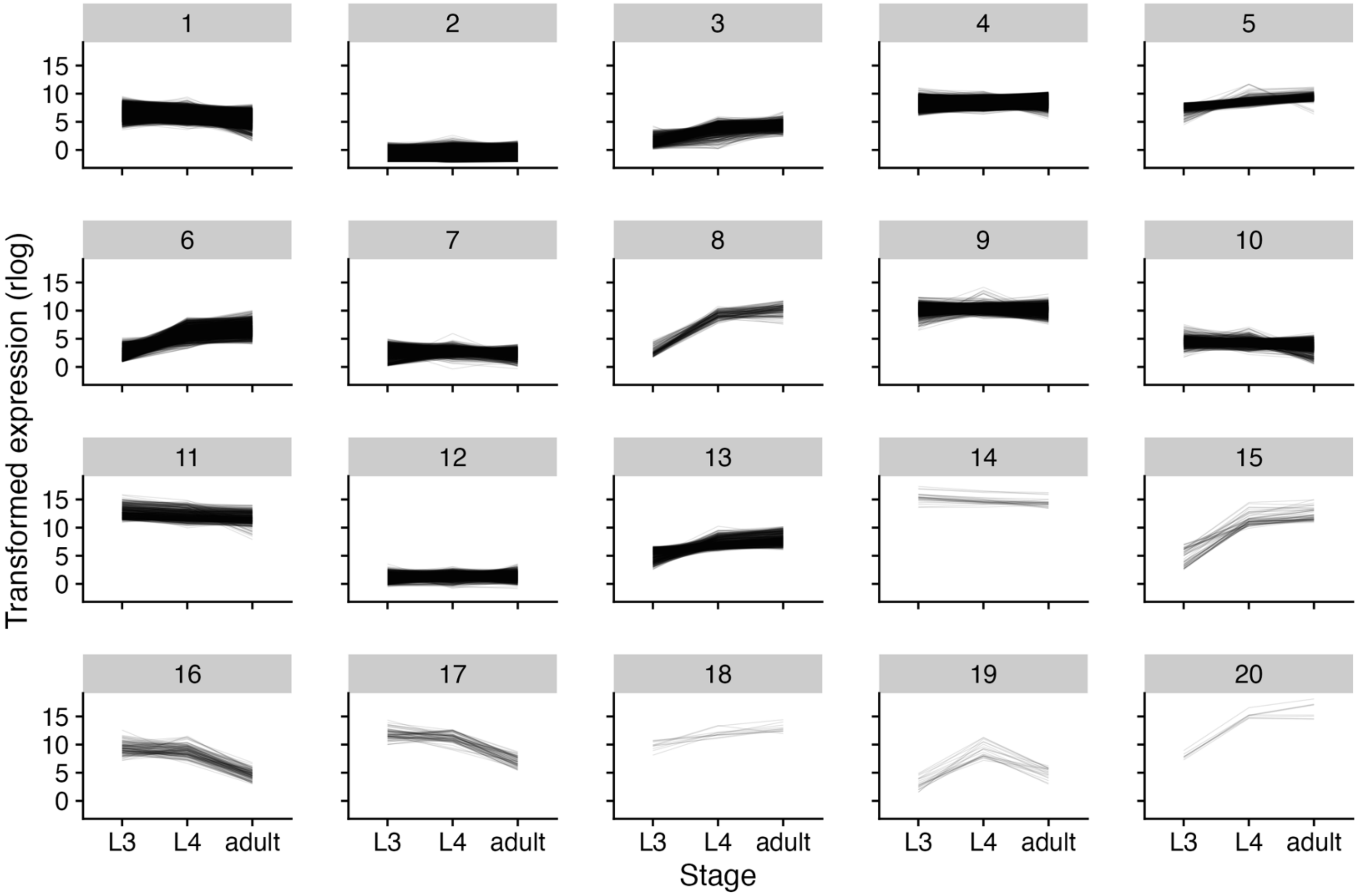

Supplemental figure 6. Clusters inferred via hierarchical clustering using all *C. inopinata* genes. Clusters were inferred with hierarchical clustering for all *C. inopinata* genes with *k*=20 clusters. For a given cluster, each line represents the average regularized-log transcript abundance for each gene at each stage in *C. inopinata*.

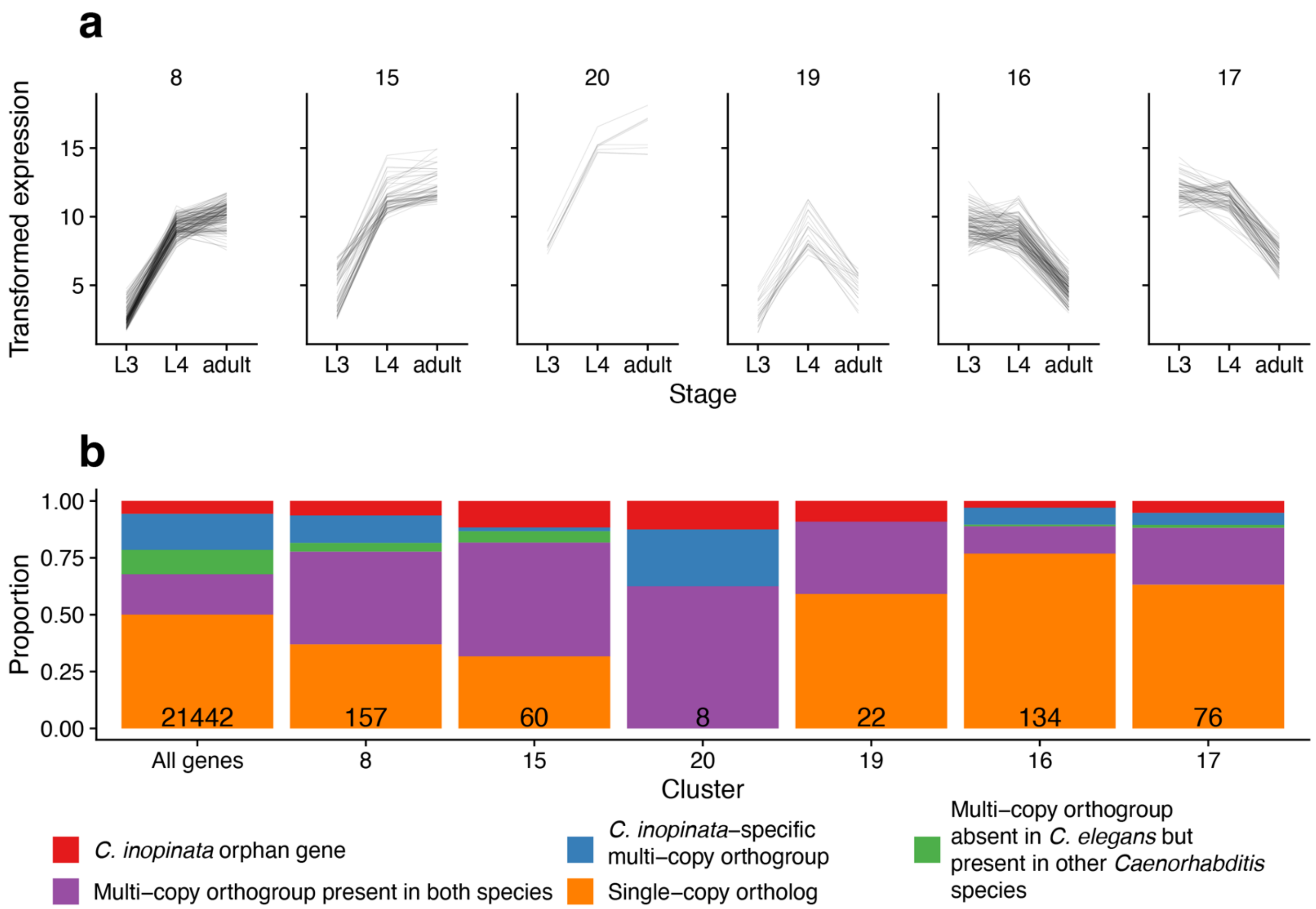

Supplemental Figure 7. Hierarchical clusters with notable transcriptional dynamics. Clusters were inferred with hierarchical clustering for all *C. inopinata* genes with *k*=20 clusters. Linear models were fit on each cluster to find those characterized by genes with significant transcriptional change over development. As most clusters revealed significant developmental dynamics, six clusters with the most striking dynamics were chosen for visualization and further analysis. (A) Transcriptional dynamics across development for six clusters. Each line represents the average regularized-log transcript abundance for each gene at each stage in *C. inopinata*. (B) Clusters classified by orthologous group categories. The number of genes in each cluster are reported at the base of each bar in the plot. Orthogroups were inferred with OrthoFinder among five *Caenorhabditis* species (*C. elegans*, *C. inopinata*, *C. remanei*, *C. briggsae*, and *C. nigoni*). Genes are categorized by the distribution of gene counts across species in the orthologous group to which the given gene belongs:

- *C. inopinata* orphan gene: a *C. inopinata* gene that clusters with *no* other genes.
- *C. inopinata*-specific multi-copy: a *C. inopinata* gene in an orthogroup containing only *C. inopinata* genes.
- Multi-copy orthogroup absent in *C. elegans* but present in other *Caenorhabditis* species: a *C. inopinata* gene in an orthogroup harboring copies in other *Caenorhabditis* species aside from *C. elegans*.
- Multi-copy orthogroup present in both species: a *C. inopinata* gene in an orthogroup harboring more than one gene copy in either *or* both of *C. inopinata* or *C. elegans*.
- Single-copy ortholog: a *C. inopinata* gene in an orthogroup harboring one gene copy in all five *Caenorhabditis* species examined.

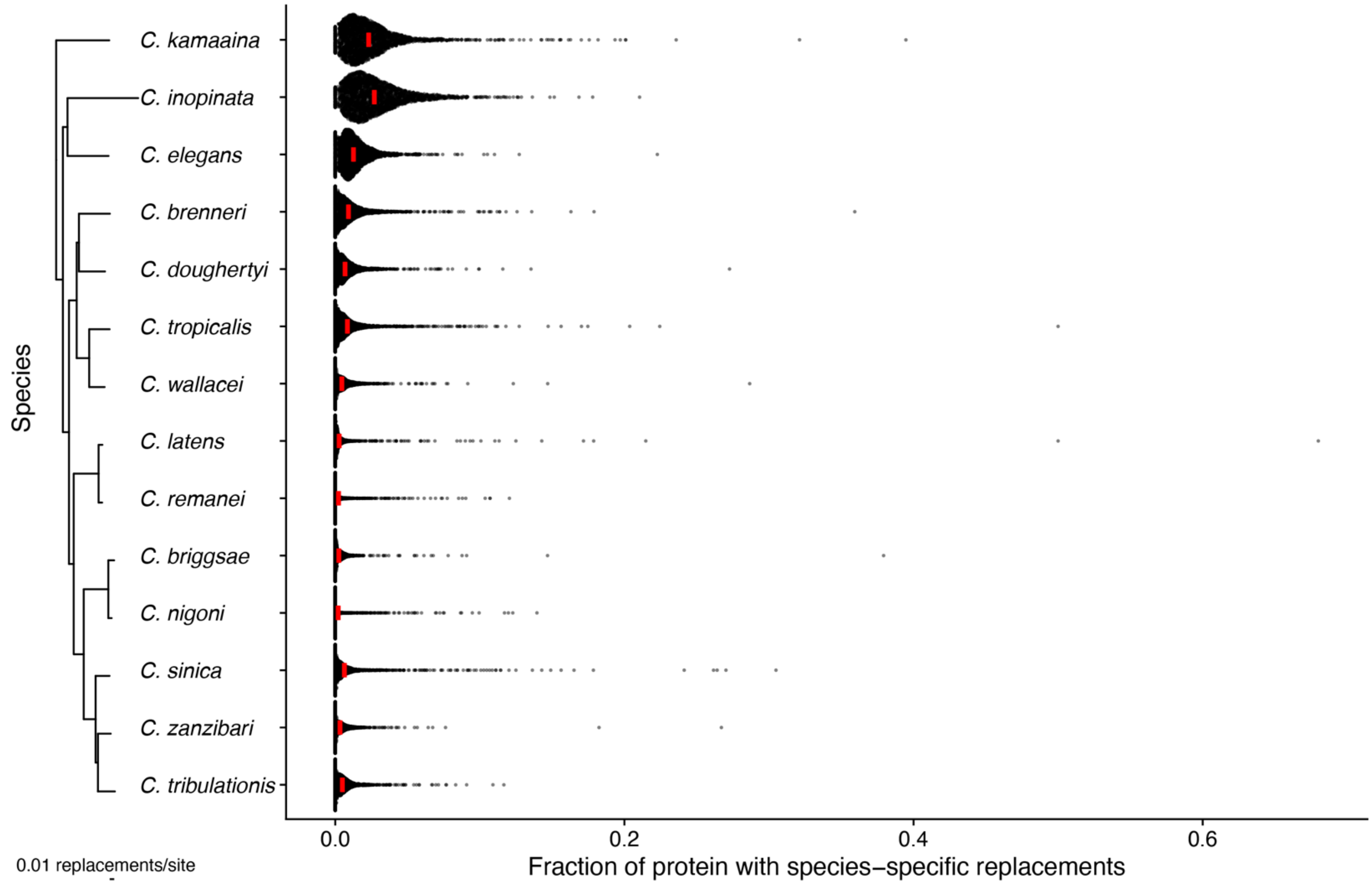

Supplemental Figure 8. Species-specific amino acid replacements. Species-specific amino acid replacements across 2,767 single-copy orthologous proteins and 14 *Caenorhabditis* species were inferred. Each point represents a protein, and plotted is the fraction of a given protein harboring species-specific amino acid replacements for a given protein. Red vertical bars represent means; sina plots are strip charts with points taking the contours of a violin plot. The phylogenetic tree plotted to the left is a trimmed version of the Bayesian tree inferred by (Stevens et al. 2019 *Evolution Letters*).

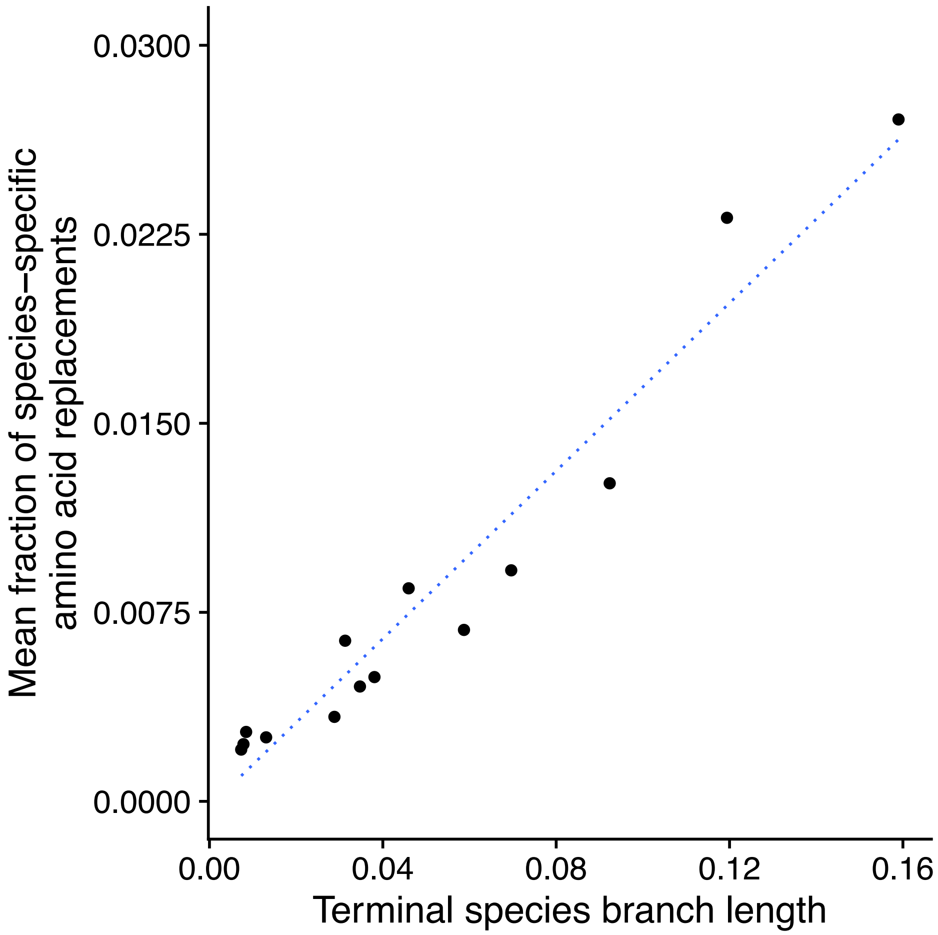

Supplemental figure 9. The number of species-specific amino acid replacements is correlated with the terminal branch length. Terminal branch lengths of the Stevens et al. 2019 *Evolution Letters* Bayesian phylogeny (associated with the 14 species analyzed) were paired with their mean protein fraction constituting species-specific amino acid replacements. These values are correlated (OLS: $y = 0.17x + 0.00021$; $p = 6.98 \times 10^{-9}$; $r^2 = 0.94$).
